# A LysR-Type Transcriptional Regulator LcrX Is Involved in Virulence, Biofilm Formation, Swimming Motility, Siderophore Secretion, and Growth in Sugar Sources in *Xanthomonas axonopodis* Pv. *glycines*

**DOI:** 10.3389/fpls.2019.01657

**Published:** 2020-01-10

**Authors:** Hanbi Park, Eunsoo Do, Minyoung Kim, Hye-Jee Park, Jongchan Lee, Sang-Wook Han

**Affiliations:** ^1^ Department of Plant Science and Technology, Chung-Ang University, Anseong, South Korea; ^2^ Department of Systems Biotechnology, Chung-Ang University, Anseong, South Korea

**Keywords:** *Xanthomonas axonopodis* pv. *glycines*, LysR type transcriptional regulators, soybean, proteomics, virulence

## Abstract

*Xanthomonas axonopodis* pv. *glycines* (*Xag*) is a Gram-negative bacterium that causes bacterial pustule disease in soybean. To acclimate to new environments, the expression of genes in bacteria is controlled directly or indirectly by diverse transcriptional factors. Among them, LysR type transcriptional regulators are well-characterized and abundant in bacteria. In a previous study, comparative proteomic analysis revealed that LysR type carbohydrate-related transcriptional regulator in *Xag* (LcrX) was more abundant in XVM2, which is a minimal medium, compared with a rich medium. However, the functions of LcrX in *Xag* have not been characterized. In this study, we generated an LcrX-overexpressing strain, *Xag*(LcrX), and the knockout mutant strain, *XagΔlcrX*(EV), to elucidate the functions of LcrX. Bacterial multiplication of *Xag*(LcrX) in soybean was significantly impaired, indicating that LcrX is related to virulence. Comparative proteomic analysis revealed that LcrX is mainly involved in carbohydrate metabolism/transport and inorganic ion transport/metabolism. Based on the results of proteomics analysis, diverse phenotypic assays were carried out. A gel electrophoresis mobility shift assay demonstrated that LcrX specifically bound to the putative promoter regions of genes encoding putative fructose 1,6-bisphosphatase and protease. Through a 96-well plate assay under various conditions, we confirmed that the growth of *Xag*(LcrX) was dramatically affected in the presence of various carbon sources, while the growth of *XagΔlcrX*(EV) was only slightly changed. Biofilm formation activity was reduced in *Xag*(LcrX) but enhanced in *XagΔlcrX*(EV). The production of siderophores was also decreased in *Xag*(LcrX) but not altered in *XagΔlcrX*(EV). In contrast, LcrX was not associated with exopolysaccharide production, protease activity, or bacterial motility. These findings provide new insights into the functions of a carbohydrate-related transcriptional regulator in *Xag*.

## Introduction

*Xanthomonas axonopodis* pv. *glycines* (*Xag*) is a Gram-negative plant pathogenic bacterium that causes bacterial pustule disease, one of the most devastating and economically important diseases in soybeans (Athinuwat et al., 2009). *Xag* infection occurs through natural openings such as the stomata and wounds on soybean leaves, and *Xag* multiplies within the intercellular spaces of mesophyll cells (Chatnaparat et al., 2012). Initial symptoms are tiny green spots observed at infection sites on leaves, which spread to the leaf surface and appear as a brown or yellow color. Finally, pustules on the leaves appear with rugged surfaces and chlorotic halos. This disease reduced the size and quantity of seeds (Narvel et al., 2001; Darrasse et al., 2013).

Transcription factors (TFs) are *trans*-acting proteins that can bind to specific *cis*-acting elements in promoter regions and regulate the initiation of transcription (Balleza et al., 2009). TFs directly or indirectly regulate expression of not only one or several genes but also numerous genes, eventually leading to the global regulation of gene expression (Browning and Busby, 2004; Geertz and Maerkl, 2010). It has been reported that approximately 51% of genes in *Escherichia coli* are directly or indirectly modulated by seven transcriptional regulators (Lrp, NarL, ArcA, FIS, IHF, FNR, and CRP) under specific conditions (Riley, 1993; Martinez-Antonio and Collado-Vides, 2003). Each TF regulates the expression of at least 50 genes, and some genes are co-regulated by different TFs. The genes regulated by the 7 TFs are involved in at least 15 biological mechanisms, including iron and carbon utilization (Zhang et al., 2005). Specifically, CRP is associated with global regulation of gene expression in *E. coli* by controlling 22 different TFs. Thus, global gene expression directly or indirectly regulated by TFs can control bacterial behaviors and phenotypic alterations.

Most TFs have two distinct functional domains: a DNA-binding domain that directly recognizes specific DNA sequences such as helix-turn-helix (HTH) domains or zinc fingers domains and an activation domain that is responsible for initiating transcription (Ohlendorf et al., 1983; Blau et al., 1996; Bouhouche et al., 2000). These types of TFs have been historically classified based on their amino acid composition in prokaryotes and eukaryotes (Mitchell and Tjian, 1989; Rivera-Gomez et al., 2011). Among them, the LysR type transcriptional regulator family (LTTR) is most abundant in prokaryotic organisms (Minezaki et al., 2005). Originally, LTTRs were known as transcriptional activators subject to negative auto-regulation, but they were recently described as global transcriptional regulators that control single or operonic gene expression as activators and repressors (Maddocks and Oyston, 2008). LTTRs contain an HTH motif in their DNA-binding domain and generally bind to the LTTR box sequence, T-N_11_-A, in the promoter regions of their regulons (Schell, 1993; Parsek et al., 1994; Aravind et al., 2005).

Some LTTRs are closely associated with the regulation of virulence-related genes. For example, MvfR regulates the expression of *pqs* operons, which are known to be regulated by quorum sensing in *Pseudomonas aeruginosa* (Deziel et al., 2005). Additionally, the virulence of *P. aeruginosa* lacking MvfR was impaired, while the mutant could still produce the quorum sensing factor *N*-acyl-l-homoserine lactone. One of the non-classical LTTRs, PA2206, is required for tolerance to oxidative stresses and contributes to global regulation in *P. aeruginosa*; a *PA2206*-knockout strain was found to be less virulent compared to the wild-type strain (Reen et al., 2013). In *Xanthomonas oryzae* pv. *oryzae* (*Xoo*), a classical LTTR, GamR, is involved in galactose metabolism and positively regulates *hrp* expression by binding to the promoters of *hrpG* and *hrpX* (Rashid et al., 2016). However, the functions of LysR type transcriptional regulators in *Xanthomonas* spp. including *Xag* remain unclear.

Previously, we reported differentially (2-fold) abundant proteins of *Xag* in rich medium and XVM2, which is a *hrp* gene inducing medium and has been widely used for identifying virulence-related genes/proteins (Wengelnik et al., 1996; Kim et al., 2003), using label-free comparative proteomic analysis (Park et al., 2017). Among them, LcrX (LysR type carbohydrate-related transcriptional regulator in *Xag*; Accession No. AOY61799) was abundantly detected in XVM2 compared with a rich medium, but its functions have not been reported. In this study, we generated a knockout mutant, *XagΔlcrX*(EV), and LcrX-overexpressing strain, *Xag*(LcrX), to investigate the functions of LcrX in *Xag*. To predict the biological mechanisms associated with LcrX, a label-free shotgun comparative proteomic analysis and clusters of orthologous groups (COGs) were used. We also confirmed the binding of LcrX through an electrophoretic mobility shift assay (EMSA) and tested the functions of LcrX using diverse phenotypic assays. Proteomic and phenotypic analyses were performed to investigate the role of LcrX in virulence, carbon utilization, iron-uptake, biofilm formation in *Xag.*

## Materials and Methods

### Bacterial Strains and Culture Conditions

Bacterial strains and plasmids used in this study are listed in **Supplementary Table S1**. *Xanthomonas axonopodis* pv. *glycines* str. 8ra (Seong et al., 2016) and mutant cells of *Xag* were grown at 28°C in tryptic soy (TS) medium (tryptic soy broth, soybean-casein digested: 30 g/L) or XVM2 medium (0.32 mM K_2_HPO_4_, 0.16 mM KH_2_PO_4_, 1 mM CaCl_2_, 5 mM MgSO_4_, 10 mM (NH_4_) _2_SO_4_, 20 mM NaCl, 0.01 mM FeSO_4_, 10 mM fructose, 10 mM sucrose, 0.03% cassamino acid) (Wengelnik and Bonas, 1996). *Escherichia coli* strains DH5α and BL21 were used as the hosts for cloning and protein expression, respectively, and were routinely cultivated in Luria Bertani medium at 37°C. Antibiotics were used for the selection of bacterial cells at final concentrations of 50 µg/ml kanamycin, 10 µg/ml gentamicin, 100 µg/ml ampicillin, 30 µg/ml cephalexin, and 100 µg/ml rifampicin for *E. coli* and *Xanthomonas* strains.

### Generation of LcrX Mutant Strains

All primers used to generate plasmids and mutants are listed in **Supplementary Table S2**. A 1301-bp DNA fragment possessing the open reading frame of *lcrX* was amplified by PCR from *Xag* genomic DNA using specific primers. The amplicons were cloned into the pGEM-T Easy vector (Promega, Madison, WI, USA) to generate pGEM-lcrX, and then this sequence was confirmed by Sanger sequencing. The middle region of pGEM-lcrX was digested with the restriction enzyme *Afe*I, and the kanamycin cassette from pUC4K was inserted, producing pGEM-lcrX::KM. The construct was introduced into the wild-type strain, *Xag* 8ra, by electroporation using a Bio Rad Micropulser™ (Bio-Rad, Hercules, CA, USA). The *lcrX* knockout mutant (*Xag∆lcrX*) was selected on TS agar plates containing kanamycin and confirmed by PCR using specific primers. The pBBR1MCS-5 vector (Kovach et al., 1995) was transferred into the *Xag*∆*lcrX* mutant, generating *Xag∆lcrX*(EV). To obtain an LcrX-overexpressing strain and complemented mutant, the open reading frame (903 bp) of *lcrX* was amplified by PCR with primer pairs containing restriction sites for *Sal*I and *Bam*HI. The amplicon was inserted into the pGEM-T Easy vector, generating pGEMLcrX-OE, which was confirmed by Sanger sequencing. To generate the overexpressing strain, *Sal*I and *Bam*HI were used to cut the *lcrX* fragment, and then cloned into pBBR1-MCS5, producing pBBR1LcrX. The expression of *lcrX* was driven by the *lac* promoter in pBBR1-MCS5. To eliminate the effect of the *lacZ* promoter in pBBR1-MCS5, we generated a promoter-less vector, pBBR1-MCS5^P^, in which 76 bp of the *lacZ* promoter region was deleted. For the complemented strain, the LcrX fragment was cloned, including the 388-bp upstream region possessing a putative promoter of *lcrX*. The fragment was cut by *Sal*I and *Bam*HI and cloned into the pBBR1MCS-5^P^ vector to generate the pBBR1LcrX^P^ construct. For the overexpressing and complemented strains, the pBBR1LcrX and pBBR1-MCS5^P^ plasmids were transferred into the wild-type and *Xag*∆*lcrX* mutant by electroporation, creating *Xag*(LcrX) and *Xag*∆*lcrX*(LcrX^P^), respectively. The transformants were confirmed by PCR using specific primers (MCS5-F and MCS5-R) (**Supplementary Table S7**).

### Virulence Assay

Virulence assays were performed by infiltration and spray methods in soybean plants, and the susceptible soybean cultivar Jinju 1 was used for testing virulence (Han et al., 2007). In the spray method, *Xag* strains were cultured in TSB medium for 48 h, harvested, and suspended in 10 mM MgCl_2_ buffer to an OD_600_ of 0.3. Soybean cultivar Jinju 1 plants were grown in a greenhouse for 15–18 days, and bacterial cell suspensions were sprayed onto fully expanded trifoliate leaves. Five soybean plants were used for each strain. After inoculation, the infected plants were kept in the humidity chamber (Relative humidity: over 90%) for the first two days. The bacterial population was measured at 0, 3, 6, and 9 days after inoculation. Infected leaves were harvested and ground using mortars and pestles with ten mM MgCl_2_. After serial dilution, the bacterial population was determined using a colony counting method onto TS agar medium containing the appropriate antibiotics. For infiltration, bacterial suspensions were prepared in 10 mM MgCl_2_ buffer to an OD_600_ of 0.3 and diluted to 1:1000. Fully expanded trifoliate leaves were inoculated using needleless syringes. The infected leaf discs were obtained using a cork borer (4 mm in diameter) at intervals of three days after inoculation. The leaf discs were ground with sterile water, and the bacterial population was measured by a colony counting method.

### Proteins/Peptide Preparation

Proteomic analysis with two sets, *Xag*(EV) vs. *Xag*(LcrX) and *Xag*(EV) vs. *Xag*∆*lcrX*(EV), were independently performed by label-free shotgun comparative proteomic analysis. Previously established protocols were used for protein extraction and peptide quantification (Park et al., 2017). Bacterial cells were grown in XVM2 medium and grown again to a final concentration, an OD_600_ of 0.5 and harvested by centrifugation at 7300 ×*g* for 15 min at 28°C. After removing the supernatants, the pellet was washed twice with 50 mM Tris-HCl (pH 7.8), re-suspended in lysis buffer (6 M Guanidine-HCl, 10 mM DTT, 50 mM Tris-HCl, pH 7.8), and disrupted with an Ultrasonic Processor (Colo Parmer, Vernon Hills, IL, USA). After sonication, the samples were centrifuged at 8,000 × *g* for 20 min at 4°C, and then the supernatants were collected. Protein concentration was determined with a BCA assay kit (Thermo Fisher Scientific, Waltham, MA, USA). The alkylation process was carried out in the presence of 100 mM iodoacetamide. Total proteins were precipitated using trichloroacetic acid, washed with acetone, and then re-suspended in 50 mM ammonium bicarbonate (pH 7.8). To digest the proteins, 5 µg of trypsin was used with 300 µg of total proteins. The digested samples were cleaned up using the Sep-Pak Vac 1cc tC18 cartridge (Waters, Milford, MA, USA). The digested protein samples were quantified with a BCA protein assay kit.

### Liquid Chromatography and Tandem Mass Spectrometry

Two microgram samples of digested peptides from three biological replicates in each strain were analyzed by split-free nano liquid chromatography (EASY-nLC II; Thermo Fisher Scientific) linked to an LTQ Velos Pro instrument (Thermo Fisher Scientific). To separate the peptides, we used a 7.5-cm column packed with MAGIG C18AQ 200A (5 µm) material (Michrom BioResources, Auburn, CA, USA). The samples were injected over 420 min with a gradient and flow rate that was maintained at 300 nL/min with water/acetonitrile gradient (solvent A: 100% H_2_O + 0.1% formic acid; solvent B: 100% acetonitrile + 0.1% formic acid; 7% B for 5 min, 35% B for 380 min, 80% B for 10 min, and final at 7% B for 25 min). To obtain the full mass spectra, six data-dependent scans were carried out over m/z 300–2,000 mass ranges. Charge state selection was allowed for 2^+^ and 3^+^ ions. Dynamic exclusion was permitted in one repeat count, 0.5 min repeat duration, and 3 min exclusion duration. The six most intense ions were sequentially selected from each full mass scan. This analysis was repeated using three biological replicates for each sample.

The Thermo proteome discoverer (ver. 1.3.0.399) combined with the SEQUEST search algorithm was used to evaluate the MS/MS spectra using the *Xag* strain 8ra database from the National Center for Biotechnology Information database. The target-decoy strategy was also used in this analysis to improve the confidence of identification (Elias and Gygi, 2007). Trypsin was assigned as the enzyme, and two missed cleavages were allowed. For all peptides, 100 ppm was used for mass accuracy, and 0.01 was used for the false discovery rate, with a probability score >20. The Scaffold 4 program (Proteome Software, Portland, OR, USA) was used to identify proteins that matched a minimum of two unique peptides. The mass spectrometry proteomics data have been deposited to the ProteomeXchange Consortium *via* the PRIDE (Perez-Riverol et al., 2019) partner repository with the dataset identifier PXD016274. Comparative analyses were carried out using peptide spectrum matches (PSMs) (Choi et al., 2008) and proteins commonly detected in the three biological replicates were selected for comparison. PSMs from selected proteins were normalized to the total PSMs from all proteins in each sample. As a comparison parameter, the average value of the PSMs from three biological replicates was used to identify differentially abundant proteins (>2-fold) in *Xag*(EV) vs. *Xag*(LcrX) or *Xag*(EV) vs. *Xag*∆*lcrX*(EV). The Student’s *t*-test was used for statistical analysis. Differentially abundant proteins were classified by clustering the orthologous group (COG) analysis (Lu et al., 2009).

### Electrophoretic Mobility Shift Assay

For expression and purification of LcrX, the pOPINM vector containing maltose-binding protein (MBP) (Berrow et al., 2007) was used. The open reading frame of *lcrX* was amplified using a specific primer set (**Supplementary Table S2**) with a Lamp Tag (Biofact, Daejeon, Korea), and the amplified fragment was cloned in pOPINM using In-Fusion Cloning technology (Takara Bio, Shiga, Japan) to prepare pOPINM-LcrX. The construct was transformed into *E. coli* BL21(DE3) cells to generate the BL21(MBP-LcrX) strain. BL21(MBP-LcrX) cells were grown in LB medium at 37°C for 16 h and then transferred into fresh LB (1:50 dilution). When the OD_600_ reached 0.6, 1 mM isopropyl-b-D-thiogalactoside was added, and the cells were incubated again for 6 h at 28°C. The cells were harvested and re-suspended in lysis buffer (20 mM imidazole, 500 mM sodium chloride, 20 mM sodium phosphate, pH 7.4). Cell lysis was conducted by adding lysozyme (a final concentration, 1 mg/ml) followed by sonication with an Ultrasonic Processor (Colo Parmer, Vernon Hills, IL, USA) 10 times (3 s on/10 s off) on ice. The total lysates were centrifuged at 12,000 × *g* for 10 min at 4°C. MBP-LcrX protein in the supernatant was purified using His GraviTrap™ columns (GE Healthcare, Little Chalfont, UK) according to the manufacturer’s protocol. The eluates were cleaned with Zeba™ spin desalting columns by the manufacturer’s protocol (Thermo Fisher Scientific) and ultrapure water. The purified MBP-LcrtX was visualized by staining with Coomassie brilliant blue R-250 (Sigma-Aldrich, St. Louis, MO, USA), and immunoblot results were confirmed using anti-His (QIAGEN, Hilden, Germany) and goat anti-rabbit IgG antibodies (Merck Millipore, Burlington, MA, USA).

To test the DNA binding activity of recombinant LcrX, electrophoretic mobility shifting assay (EMSA) was performed as previously described with minor modification (Imperi et al., 2010). The DNA probes for EMSA were generated by PCR using the specific primers listed in **Supplementary Table S2**. All probes were designed to amplify 210 bp preceding the start codon The binding reaction with the specific DNA probe was carried out in binding buffer (20 mM Tris-HCl (pH 7.5), 1 mM DTT, 0.1 mM EDTA, 10% glycerol, 10 mg/ml bovine serum albumin, 50 mM KCl, 10 mM MgCl_2_, 5 μg/ml poly di-dc). The reaction mixtures containing recombinant LcrX and ^32^P-end labeled double-stranded probe DNA were incubated at room temperature for 20 min, and the samples were subjected into 6% native-polyacrylamide gel electrophoresis that had been pre-electrophoresed for 30 min in 1X TBE (89 mM Tris, 89 mM borate, and 2 mM EDTA) at room temperature. The DNA-protein complex was separated by electrophoresis at room temperature and 130 V for 1 h. The gel was dried and exposed to a phosphor screen (PerkinElmer, Waltham, MA, USA), and bands were detected with a Packard Cyclone Phosphor Imager (PerkinElmer).

### Quantitative Reverse Transcription PCR

Gene expression was evaluated by quantitative reverse transcription PCR (q-RT-PCR). All oligonucleotide primers used for q-RT-PCR are shown in **Supplementary Table S2**. Bacterial cells were grown in media and harvested at an OD of 0.6. The total RNA was extracted with a High Pure RNA Isolation Kit (Roche, Mannheim, Germany), and cDNA synthesis was performed following the protocol of the RevertAid First Strand cDNA Synthesis Kit (Thermo Fisher Scientific). The 16S ribosomal RNA was employed as a reference gene for normalization. q-RT-PCR was performed using an IQ™ SYBR Green Supermix (Bio-Rad) on a CFX connect™ (Bio-Rad). The experiment was repeated at least twice with three replicates. The 2^−ΔΔCt^ method was used to calculate gene expression levels.

### Extracellular Protease Activity

Extracellular protease activity was examined as previously described (Sole et al., 2015) with slight modifications. *Xag* strains were grown in TSB medium, washed with sterile water, and then adjusted to an OD_600_ of 1.0. Three microliters of bacterial suspension were dropped onto NYG plates containing 1% skim milk (BD Biosciences, Franklin Lakes, NJ, USA) and 1% agar, and then incubated at 28°C for 7 days. The clear halo zone was measured.

### GEN III Microplate Assay

The growth ability of the *Xag* strain under 94 different conditions was investigated in a GEN III microplate (Biolog™, Hayward, CA, USA) using the provided protocol with slight modifications. The GEN III microplate is a part of a phenotype microarray system consisting of panels of 96 wells containing diverse carbon sources and chemical substrates in each well (94 different conditions). *Xag* strains were grown in TSB and washed three times with sterile water. Bacterial cells were adjusted to an OD_590_ of 0.02 in incubating fluid (Biolog™). One hundred microliters of bacterial suspensions were mixed in each well and incubated at 28°C. Growth was then determined by measuring the absorbance at 590 nm after 24 and 48 h using a Spectramax 190 microplate reader.

### Bacteria Growth in Rich and Minimal Medium

To determine of Bacteria growth in rich and minimal condition, TSB and XVM2 medium were used. *Xag* strains were incubated at 28°C and then adjusted to an OD_600_ of 0.3. After washing twice, bacterial suspensions were diluted to an OD_600_ of 0.003 with TSB and XVM2. Growth was measured with a UV spectrophotometer at 600 nm at 24-h intervals for 72 or 120h.

### Bacteria Growth in Different Sugar Sources

To confirm the cell growth ability in the presence of sugar sources as the sole carbon source, M9 medium was used in the supplement of 0.4% glucose, sucrose, and fructose. *Xag* cells were grown in TSB, washed twice with sterile water, and re-suspended in M9 medium. The bacterial suspension was adjusted to an OD_600_ of 0.001 in M9 medium, and growth was measured with a UV spectrophotometer at 600 nm at 24-h intervals for 96 h.

### Biofilm Formation and Exopolysaccharide Production Assay

Biofilm formation was determined in XVM2 liquid medium as described previously with slight modifications (Park et al., 2014). *Xag* strains were grown for 2 days in TSB medium and washed using sterile water. The bacterial suspension was adjusted to an OD_600_ of 0.3 and diluted to 1:1000 in XVM2 liquid media. The bacterial suspension was incubated in a 96-well PVC (polyvinyl chloride) plate at 28°C for 8 days. After incubation, the supernatant was carefully eliminated, and attached bacterial cells were stained with 0.1% crystal violet. The stained cells were solubilized in 95% ethanol for 20 min, and absorbance was measured at 590 nm with a Spectramax 190 microplate reader (Molecular Devices, Sunnyvale, CA, USA) with 14 biological replicates.

Exopolysaccharide (EPS) production was evaluated as previously described with slight modifications (Bae et al., 2018b). *Xag* strains were grown to an OD_600_ of 0.1 in 5 ml XVM2 for 5 days at 28°C. The bacteria suspension was centrifuged at 10,500 ×*g* for 3 min at 28°C, and 400 µl of the supernatant was added in 1.2 ml of 95% ethanol and stored overnight at −20°C. The samples were centrifuged, and then the pellets were dried at room temperature. The pellets were added to 1 ml sterile water and mixed with 1 ml of 5% aqueous phenol and 5 ml of H_2_SO_4_ and EPS was quantified by measuring the absorbance of the mixtures at 488 nm.

### Swimming and Swarming Motility Assays

The swimming and swarming motility assays were performed as described previously with slight modifications (Bae et al., 2018a). Swarming and swimming and motilities were determined on an XVM2 plate containing 0.3% agar. *Xag* strains were grown for 2 days in TSB medium and washed with sterile water. The bacterial suspension was adjusted to an OD_600_ of 0.3, and 3 µl of each bacterial cell suspension was spotted on the surface for swarming and placed into in the middle of XVM2 plates for swimming by stabbing using tips onto the center and incubated at 28°C for 3 days. The diameter of the colony occupied by each strain was measured.

### Chrome Azurol S (CAS) Assay

Siderophore production was assessed by performing a chrome azurol S assay as described previously with slight modifications (Park et al., 2019). XVM2-CAS-agar was used to create an iron-rich condition. For the iron-limited condition, 2, 2′-bipyrudyl (BP) was added at a final concentration of 100 μM (XVM2-CAS-BP). *Xag* strains were grown on TS agar medium for 2 days, harvested, washed three times with sterile water, and adjusted to an OD_600_ of 0.3. Three microliters of bacterial suspension were dropped onto the XVM2-CAS or XVM2-CAS-BP plate. The diameters of the halo zone and colony sizes observed on the plates were measured after incubation at 3 days.

### In *Silico* Modeling in LcrX

To predict the structure of LcrX, the deduced amino acid (aa) sequence of LcrX was evaluated with the I-TASSER server (Roy et al., 2010), and the PDB file for LcrX was obtained. PyMOL software (Molecular Graphics System, San Carlos, CA, USA) was used to generate the predicted three-dimensional structure of LcrX.

### Statistical Analysis

The statistical significance of quantitative data was identified by Student’s *t*-test and one-way analysis of variance with Tukey’s multiple comparison using SPSS 12.0K software (SPSS, Inc., Chicago, IL, USA). A P-value of less than 0.05 was considered to indicate a significant difference.

## Results

### LcrX Is a LysR Type Transcriptional Regulator

**Figure 1:**
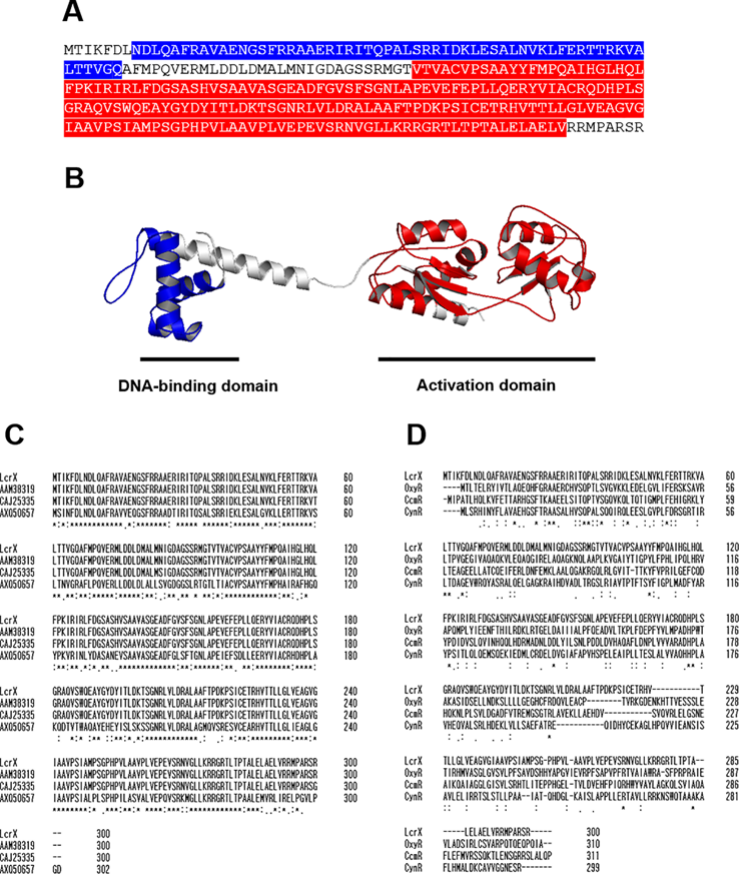
Predicted three-dimensional structure and sequence alignment of LcrX. **(A)** Deduced amino acid sequence of LcrX where blue and red boxes indicate DNA-binding domain and LysR activation domain, respectively. **(B)** Predicted 3D structure obtained using the protein modeling server I-TASSER and PyMOL program. **(C)** Comparison of amino acid sequences of LcrX with its homologs in *Xanthomonas* spp. and *Stenotrophomonas* spp. using the ClustalOmega program. CAJ25335 from *X. campestris* pv. *vesicatoria* str. 85-10, AAM38319 from *X. citri* pv. *citri* str. 306, AXQ50657 from *Stenotrophomonas rhizophila* str. GA1 **(D)** Comparison of amino acid sequences of LcrX with LysR type transcriptional regulators whose functions were previously characterized. OxyR from *Pseudomonas aeruginosa* str. PAO1, CcmR from *Synechococcus* sp. str. PCC 7002, and CynR from *Escherichia coli* str. K-12. ‘*’, ‘.’, and ‘:’ indicate most conserved residues, semi-conserved sequence, respectively.

LysR type transcriptional regulators (LTTRs) possess two domains, a DNA-binding domain showing HTH motifs and an activation domain for transcription initiation (Johnson et al., 1993; Yura et al., 1993). Analysis of the deduced amino acid sequence of LcrX revealed two conserved domains, a DNA-binding domain (HTH; 8–67 aa) and an activation domain (96–262 aa) (**Figure 1A**), indicating that LcrX belongs to the LTTR family. The predicted 3D structure of LcrX by I-TASSER (Roy et al., 2010) and PyMOL clearly revealed two distinct domains (**Figure 1B**), supporting that LcrX is a putative LTTR. Next, we compared the sequence of LcrX at the amino acid level with putative LTTRs in other bacteria (**Figure 1C**). High homology was observed between LcrX and CAJ25335 (297/300, 99%), AAM38319 (300/300 100%), and AXQ50657 (211/300, 70%) in *X. campestris*, *X. citri*, and *Stenotrophomonas rhizophila*, respectively (**Figure 1C**), indicating that LcrX is conserved in *Xanthomonas* spp. and closely related genera. Finally, the deduced amino acid sequence of LcrX was compared to those of other LTTRs whose functions have been characterized (Lamblin and Fuchs, 1994; Wei et al., 2012). However, LcrX exhibited low homology with CynR related to the cyanate metabolism (78/300, 26%) (Lamblin and Fuchs, 1994), OxyR involved in an oxidative stress (62/300, 20%), (Ochsner et al., 2000), and CcmR associated with the inorganic carbon concentrating mechanism (67/300, 22%) (Woodger et al., 2007) from *E*. *coli*, *P*. *aeruginosa*, and *Synechococcus*, respectively (**Figure 1D**), indicating that LcrX has distinct functions from the three LTTRs.

### Overexpression of LcrX Reduced the Virulence of *Xag*

**Figure 2:**
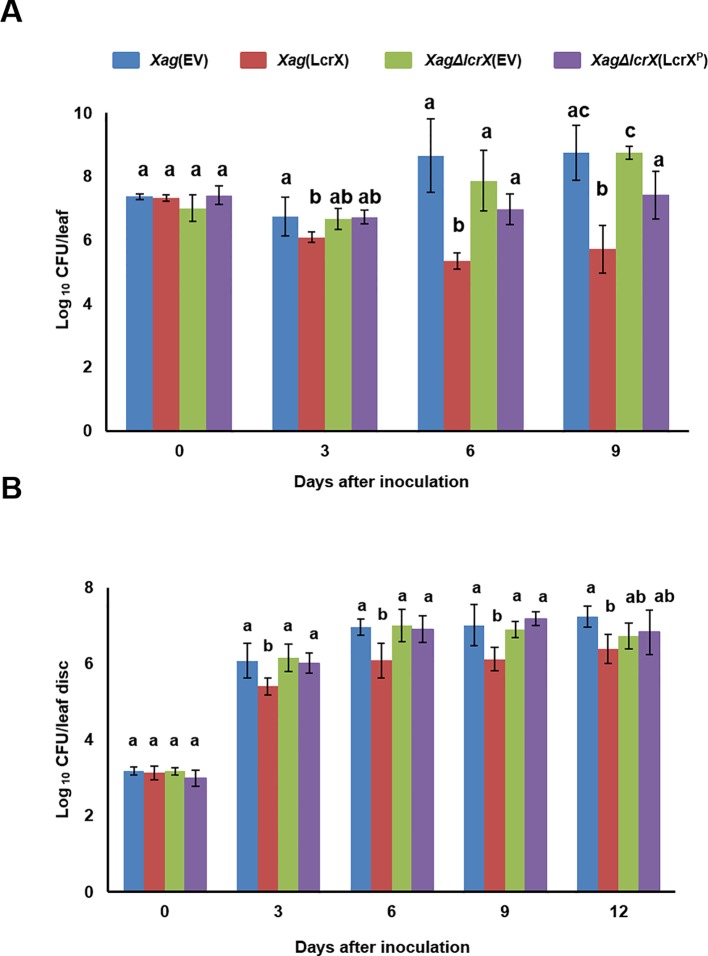
Growth test of *Xag*(EV), *Xag*(LcrX), *XagΔlcrX*(EV), and *XagΔlcrX*(LcrX^P^) using two different inoculation methods on soybean leaves. Leaves of soybean cv. Jin-ju were inoculated by **(A)** the spray method and **(B)** infiltration. In the spray method, bacterial suspensions were adjusted to an OD_600_ of 0.3 in 10 mM MgCl_2_ and sprayed onto fully expanded trifoliate leaves. In the infiltration test, bacterial suspensions were adjusted to an OD_600_ of 0.3 and serially (10^−3^) diluted, and then infiltrated with needleless syringes. Bacterial populations were determined using the colony counting method at 3-day intervals. Error bars represent the standard deviation of five biological replicates. The different letters on the bars represent significant differences by one-way ANOVA (p < 0.05). This experiment was repeated at least three times.

Because LcrX was abundantly detected in XVM2 medium (Park et al., 2017), we examined the effect of LcrX on the virulence of *Xag* in susceptible soybean using the wild-type strain carrying an empty vector *Xag*(EV), LcrX-overexpressing strain *Xag*(LcrX), *lcrX*-knockout mutant carrying an empty vector *Xag*∆*lcrX*(EV), and complemented strain *Xag*∆*lcrX*(LcrX^P^). Expression of LcrX in *Xag*(LcrX) and *Xag*∆*lcrX*(LcrX^P^) was driven by the Lac promoter and putative native promoter of *lcrX*, respectively. The virulence of the four strains was examined using two different methods, spray inoculation (**Figure 2A**) and infiltration with needleless syringes on the leaves (**Figure 2B**). In both methods, bacterial growth in the leaves of soybean showed similar patterns. In the spray method (**Figure 2A**), the population of *Xag*(LcrX) was significantly lower than that of *Xag*(EV) at 3 (29-fold), 6 (>500-fold), and 9 (> 140-fold) days after inoculation. However, the bacterial growth of *Xag*∆*lcrX*(EV) and *Xag*∆*lcrX*(LcrX^P^) was not significantly different from that of *Xag*(EV). The disease symptoms by *Xag*(LcrX) were significantly reduced in infected leaves compared with *Xag*(EV) and *Xag*∆*lcrX*(EV) (**Supplementary Figure S1A**), but *Xag*(LcrX) showed typical pustule symptoms like other three strains (**Supplementary Figure S1B**). The difference in population between *Xag*(LcrX) and *Xag*(EV) in the infiltration method was lower than that in spray inoculation. *Xag*(LcrX) also showed a reduced population at 3 (5-fold), 6 (4-fold), 9 (8-fold), and 12 days after inoculation (5-fold) compared to *Xag*(EV). *Xag*∆*lcrX*(EV) and *Xag*∆*lcrX*(LcrX^P^) showed similar growth patterns as *Xag*(EV). However, disease symptoms caused by the infiltration were not very different among *Xag* strains (**Supplementary Figure S1C**). These results indicate that overexpression of LcrX negatively affects the virulence of *Xag* in soybean, regardless of the inoculation method.

### Comparative Proteomic Analysis of LcrX

**Figure 3:**
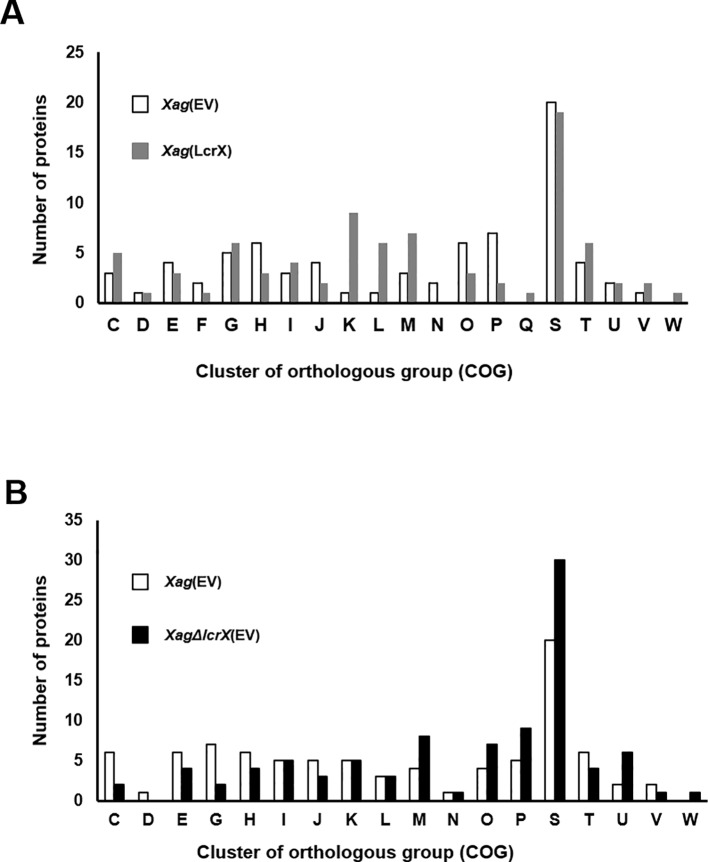
Clusters of orthologous groups (COG) of proteins whose expression is regulated by LcrX. Bar graphs indicate **(A)** the COG category of 75 and 78 proteins, which were more (over 2-fold) abundant in *Xag*(EV) and *Xag*(LcrX), respectively and **(B)** COG category of 84 and 90 proteins, which were more (over 2-fold) abundant in *Xag*(EV) and *XagΔlcrX*(EV), respectively. C, Energy production and conversion; D, Cell cycle control and mitosis; E, Amino acid metabolism and transport; F, Nucleotide metabolism and transport; G, Carbohydrate metabolism and transport; H, Coenzyme metabolism; I, Lipid metabolism; J, Translation; K, Transcription; L, Replication and repair; M, Cell wall/membrane/envelop biogenesis, N, Cell motility; O, Post-translational modification, protein turnover, chaperone functions; P, Inorganic ion transport and metabolism; Q, Secondary structure; R, General functional prediction only; S, Function unknown; T, Signal transduction; U, Intracellular trafficking and secretion; V, Defense mechanisms; W, Extracellular structures.

In a previous study, we confirmed that LcrX was abundantly in XVM2 medium rather than in TSB. Therefore, we carried out q-RT-PCR to evaluate the expression of the LcrX transcript. As a result, *lcrX* expression was much higher (9.8-fold) in XVM2 compared to in TSB (**Supplementary Figure S2**). Because overexpression of LcrX clearly contributes to reduced virulence, we predicted the biological mechanisms related to LcrX in *Xag* using a label-free shotgun comparative proteomic analysis combined with COG analysis. In the comparison of *Xag*(EV) and *Xag*(LcrX), a total of 1178 and 1119 proteins were commonly detected in the three biological replicates, respectively (**Supplementary Table S3**). These proteins were subjected to comparative analysis, which showed that 75 and 78 proteins were differentially (over 2-fold) abundant in *Xag*(EV) and *Xag*(LcrX), respectively (**Supplementary Tables S4** and **S5**). The LcrX protein was mainly detected in *Xag*(LcrX), indicating that *Xag*(LcrX) is indeed an LcrX-overexpressing strain (**Supplementary Table S5**). COG classification was used to group these differentially abundant proteins by similar functions (**Figure 3**). Proteins belonging to the G (carbohydrate metabolism and transport), K (transcription), L (replication, recombination, and repair), M (cell wall/membrane/envelope biogenesis), and T (signal transduction mechanisms) groups were abundantly detected in *Xag*(LcrX) (**Figure 3A**). Additionally, proteins categorized in the H (coenzyme transport and metabolism), J (translation), O (post-translational modification, protein turnover, and chaperones), and P (inorganic ion transport and metabolism) groups were highly detected in *Xag*(EV) (**Figure 3A**). Notably, the N (cell motility) group was not found in *Xag*(LcrX). Interestingly, at least 8 proteins associated with carbohydrate metabolisms, such as two glycerophosphodiester phosphodiesterases, xylosidase, mannosidase, galactonate dehydratase, galactose dehydrogenase, sorbosone dehydrogenase, and alcohol dehydrogenase, were abundantly detected in *Xag*(LcrX) (**Supplementary Table S5**). Moreover, five proteins involved in iron uptake (bacterioferritin, ferrous iron transporter B, and three TonB-dependent receptors) were more abundant in *Xag*(EV) than in *Xag*(LcrX) (**Supplementary Table S4** and **S5**). Collectively, these results suggest that LcrX is closely involved in carbohydrate metabolism and iron uptake within *Xag*.

Next, we compared proteins from *Xag*(EV) to those from *Xag*∆*lcrX*(EV). This experiment was carried out independently of the comparison of *Xag*(EV) and *Xag*(LcrX). In LC-MS/MS analysis, 1091 and 1097 proteins were shared in the three biological replicates of *Xag*(EV) and *Xag*∆*lcrX*(EV), respectively (**Supplementary Table S3**). A total of 174 proteins were differentially expressed between *Xag*(EV) and *Xag∆lcrX*(EV), and 84 and 90 proteins were more abundant (over 2-fold) in *Xag*(EV) and *Xag*∆*lcrX*(EV), respectively (**Supplementary Table S6** and **S7**). COG analysis showed that the expression of proteins categorized by the M (cell wall/membrane/envelop biogenesis), O (post-translational modification, protein turnover, and chaperones), P (inorganic ion transport and metabolism), and U (intracellular trafficking, secretion, and vesicular transport) groups was highly affected in *Xag*∆*lcrX*(EV) (**Figure 3B**). Proteins belonging to the C (energy production and conversion) and G (carbohydrate metabolism and transport) groups were abundant in *Xag*(EV). Similar to in the *Xag*(EV) vs *Xag*(LcrX) comparison, seven proteins involved in carbohydrate metabolism (succinate dehydrogenase, pyruvate dehydrogenase, alcohol dehydrogenase, glycosyl hydrolase, glycogen debranching enzyme, glucosidase, and amylase), and six proteins involved in iron uptake (ferrochelatase and five TonB-dependent receptors) were detected (**Supplementary Table S6** and **S7**).

### LcrX Directly Bound to Putative Promoters of Fructose 1,6-Bisphosphatase and Protease

**Figure 4:**
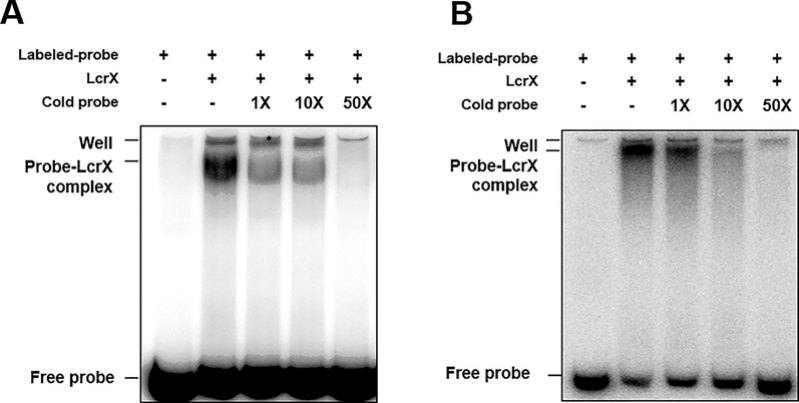
Electrophoretic mobility shift assay of LcrX. EMSA was performed to determine between LcrX and putative promoter probes from **(A)** AOY60939 (fructose 1,6-bisphosphatase) and **(B)** AOY62452 (protease) labeled with [γ -^32^P]. The binding reaction was performed using 0.5 μM of recombinant LcrX with 20 ng of labeled probes. Cold probes, 1X (20 ng), 10X (200 ng), and 50 X (1,000 ng), were added for a competition assay.

If LcrX is a transcriptional regulator, it may regulate the expression of genes by binding to the promoters of its target genes. To examine whether LcrX and putative promoters directly interact, an EMSA was carried out. Despite several attempts, successful purification of the native and soluble LcrX in *E. coli* BL21 was not possible because expressed LcrX formed inclusion bodies. To increase solubility, we generated LcrX fused to MBP and successfully purified native and soluble MBP-LcrX with a size of approximately 75 kDa (**Supplementary Figure S3**). Next, we selected putative targets of LcrX in the EMSA using the following categories: I) abundance of proteins in comparative proteomics, II) COG classification combined with phenotypic alteration, and III) the numbers of PSMs detected from LC-MS/MS analysis. As a result, we selected eight genes, AOY61777 (monothiol glutaredoxin and Grx4 family), AOY60939 (fructose 1,6-bisphosphatase, FBP), AOY62387 (TonB-dependent receptor), AOY62229 (hypothetical protein), AOY62452 (protease), AOY64564 (bacterioferritin), AOY64093 (glycine C-acetyltransferase), and LcrX. We selected LcrX because some LTTRs are auto-regulated (Lindquist et al., 1989; Schell, 1993). For the EMSA, we amplified the putative promoter region, which is 210 bp from the start codon, from each gene using specific primer sets. Among the eight candidates, recombinant LcrX bound to the putative promoters of fructose 1,6-bisphosphatase and protease, but not to the other six genes, including LcrX (**Supplementary Figure S4**). Thus, LcrX is not auto-regulated. To confirm whether LcrX specifically bound to the two promoter regions, unlabeled probes were used as competitors (**Figure 4**). As the amounts of unlabeled probes increased, probe-LcrX bands gradually decreased in both cases (**Figure 4**), demonstrating that LcrX binds directly to the putative promoters of FBP and protease. Therefore, we also evaluated the transcripts of both fructose 1,6-bisphosphatase and protease by q-RT-PCR. As expected, the gene expression of FBP and protease was consistent with the pattern of protein abundance (**Supplementary Figure S5**). The transcripts of both genes were lower and higher in *Xag*(LcrX) and *Xag*∆*lcrX*(EV), respectively, compared to in *Xag*(EV). Taken together, these data indicate that LcrX negatively regulates the expression of FBP and protease.

### LcrX Is Involved in the Growth of *Xag* Under Diverse Conditions

**Figure 5:**
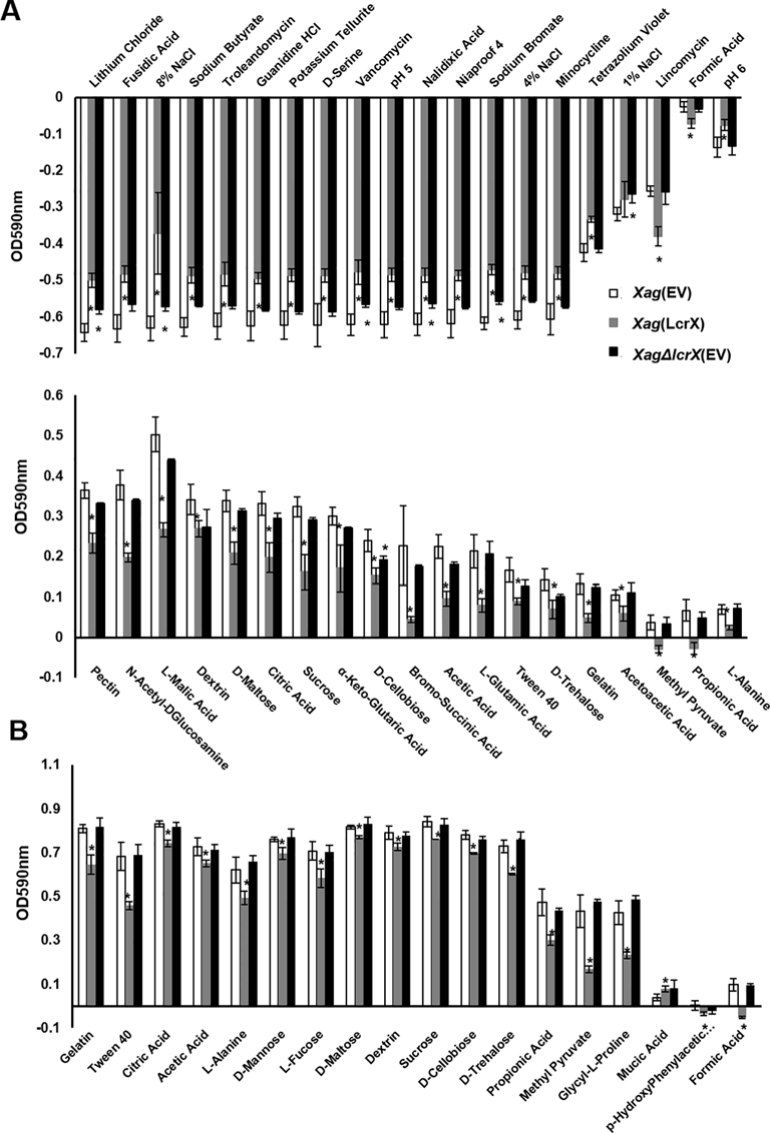
Comparison of growth of *Xag*(EV), *Xag*(LcrX), and *XagΔlcrX*(EV) under 94 different conditions. The GEN III plate, which contains 71 different carbon sources and 23 different chemicals was used for this assay. The bacterial suspensions were adjusted at OD_590_ of 0.02, incubated in GEN III plate for 2 days, and then measured at 590 nm. The graph represents different sources in which the growth of *Xag*(LcrX) and/or *XagΔlcrX*(EV) was different from that of *Xag*(EV) at **(A)** 24 h and **(B)** 48 h after incubation. White, gray, and black indicate *Xag*(EV), *Xag*(LcrX), and *XagΔlcrX*(EV), respectively. The plus and minus values indicate more and less growth compared with the controls, respectively. Bars in the graph represent the means of three biological replicates with the standard deviation and asterisks indicate significant differences (*t* test, p < 0.05).

Because deduced amino acid sequences of protease reveal that the proteins is not a cytoplasmic protein, we examined an extracellular protease activity in four strains, but the activity did not differ under the conditions used (**Supplementary Figure S6**). Therefore, we focused on the growth ability of *Xag* under various conditions including in the presence of diverse sugar sources. We compared the growth ability of *Xag*(EV), *Xag*(LcrX), and *Xag∆lcrX*(EV) using a GEN III plate containing 71 different carbon sources, and 23 different chemicals or conditions (**Figure 5**). After incubation for 24 h, the growth of *Xag*(LcrX) was significantly different from that of *Xag*(EV) under 38 different conditions. Among the 38 different conditions, 21 were carbon sources and 17 were chemical substrates (**Figure 5A**). However, *Xag∆lcrX*(EV) showed differences in growth under only 7 conditions, one carbon source, and 6 chemical substrates. After incubation for 48 h in the GEN III plate, *Xag*(LcrX) showed significantly different growth in 18 carbon sources but not in any chemical substrates (**Figure 5B**). Interestingly, the growth of *Xag*∆*lcrX*(EV) was not significantly different from that of *Xag*(EV) under all conditions after incubation for 48 h.

### LcrX Controls the Growth of *Xag* in the Presence of Diverse Sugar Sources

**Figure 6:**
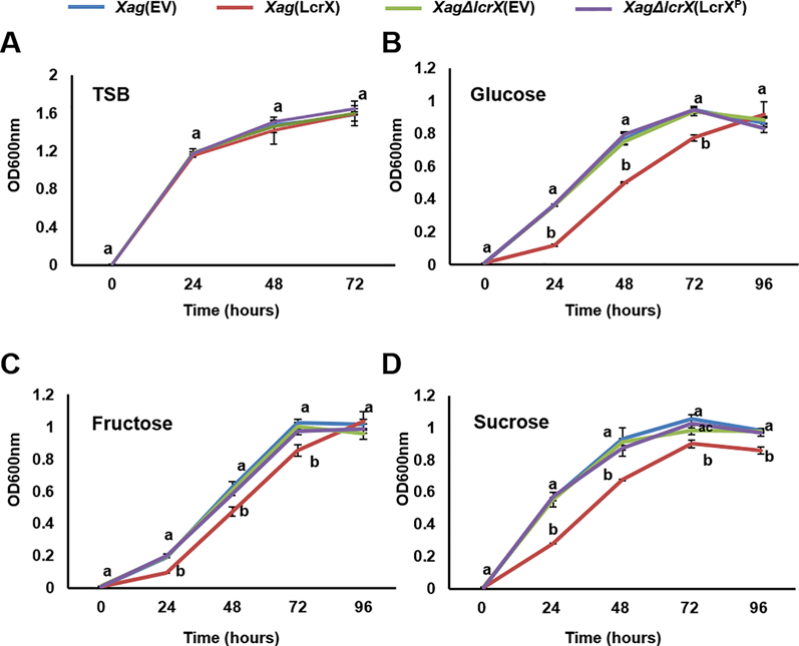
Growth of *Xag* strains on TSB and M9 minimal medium containing different sugar sources. *Xag* strains were grown at 28°C in TSB **(A)**, and modified M9 medium with 0.4% glucose **(B)**, fructose **(C)**, or sucrose **(D)**. The bacterial cells were measured at 24-h intervals at an OD of 600 nm. Different letters on error bar graph indicate significant differences by one-way ANOVA (p < 0.05). This experiment was repeated at least three times.

Proteomic analysis using the EMSA and GENIII plate assay suggested that LcrX directly regulates the expression of FBP and is closely related to the utilization of different types of sugar sources. We further investigated the growth of four strains in the presence of fructose, glucose, or sucrose as the sole carbon source. In TSB, there was no difference in the growth of the four strains (**Figure 6A**), indicating that LcrX is not associated with bacterial multiplication in the nutrient-rich condition. However, *Xag*(LcrX) showed slow growth compared to *Xag*(EV), while *XagΔlcrX*(EV) and *Xag∆lcrX*(LcrX^P^) displayed similar patterns as *Xag*(EV) when supplemented with fructose, glucose, or sucrose (**Figures 6B**–**D**). In the presence of glucose and fructose, the population of *Xag*(LcrX) was reduced compared to the other strains at 24, 48, and 72 h after incubation (**Figure 6B** and **6C**). In sucrose, the growth of *Xag*(LcrX) was significantly lower than those of the three strains (**Figure 6D**). These data indicate that the functions of LcrX are associated with bacterial growth in the presence of sugar sources.

### LcrX Is Related to Biofilm Formation But Not EPS Production and Motility

**Figure 7:**
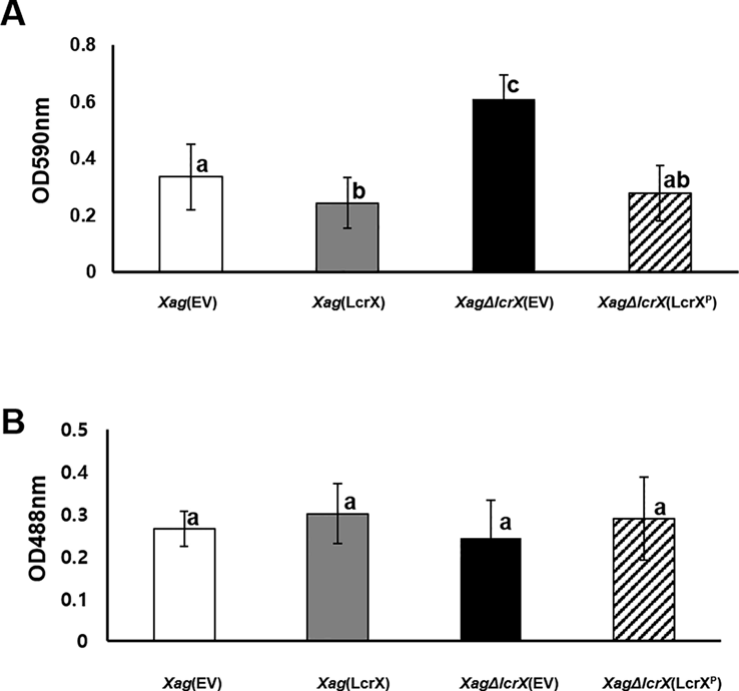
Biofilm formation and exopolysaccharide (EPS) production in *Xag*(EV), *Xag*(LcrX), *XagΔlcrX*(EV), and *XagΔlcrX*(LcrX^P^). **(A)** Biofilm formation ability in *Xag* strains was evaluated by polyvinyl chloride plate (PVC) plate assay. *Xag* strains were incubated in the PVC plates for eight days and cells attached to the surface were stained with crystal violet and resolved in 95% ethanol. Biofilm formation was enumerated using a spectrophotometer at 590 nm. Error bars in the graph indicate the means of 14 biological replicates with the standard deviation. **(B)** EPS production by *Xag* strains was quantified by the phenol-sulfuric acid method. *Xag* strains were incubated in TSB for 2 days and in XVM2 for 4 days, and then EPS was quantified by a phenol-sulfuric acid method. EPS was quantified with a spectrophotometer at 488 nm. Bars indicate the means of three biological replicates with the standard deviation. Different letters represent significant differences by one-way ANOVA (p < 0.05). This experiment was repeated at least three times.

**Figure 8:**
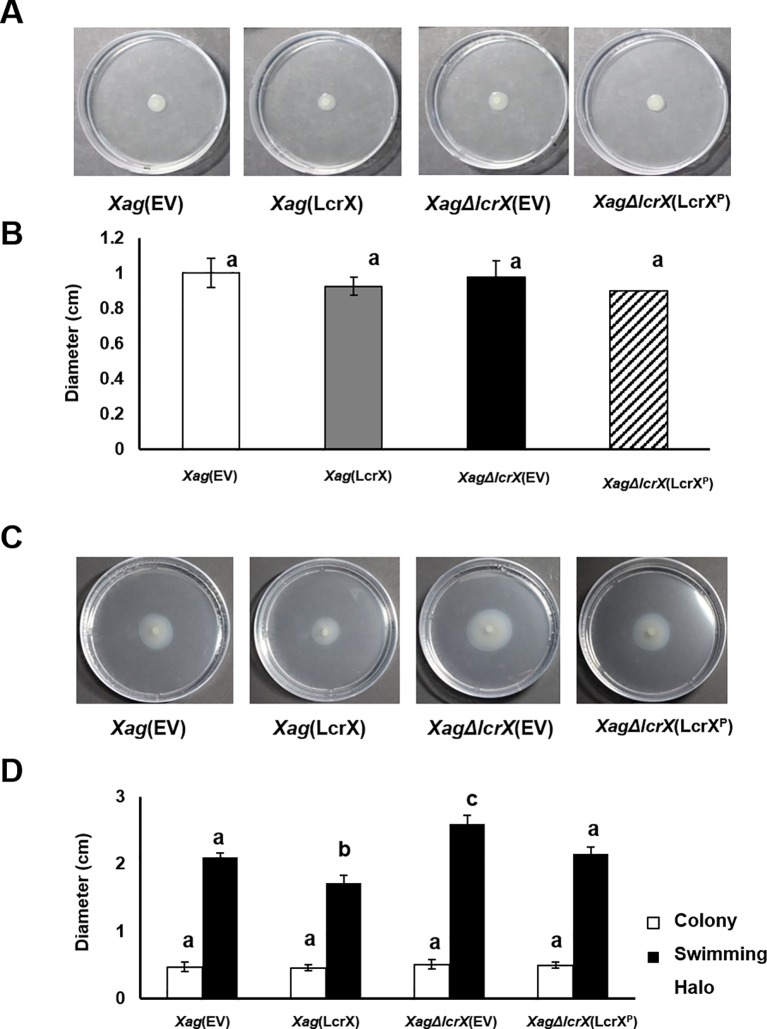
Swimming and swarming motility of *Xag* strains. Three microliters of bacterial suspension (OD_600_ of 0.3) were spotted onto the semi-solid medium (0.3% agar) for swarming **(A** and **B)** or positioned in to the middle of the medium by stabbing **(C** and **D)**. Photographs of the motilities **(A** and **C)** were taken, and the expansion of colonies and colony halos **(C** and **D)** was measured at 3 days after incubation. Bars in the graph indicate the means of five biological replicates with the standard deviation. Different letters represent significant differences by one-way ANOVA (p < 0.05). This experiment was repeated at least five times.

Biofilm formation is an important virulence factor by which bacteria protect themselves against diverse environmental stresses (Costerton et al., 1995). Polysaccharides and proteins are the main components in bacterial biofilms (Nielsen et al., 1997). Proteomic analyses revealed diverse proteins associated with carbohydrate metabolism in both sets [*Xag*(EV) vs. *Xag*(LcrX)] and [*Xag*(EV) vs. *Xag*∆*lcrX*(EV)] (**Supplementary Tables S4**–**S7** and **Figure 3**) and LcrX was involved in carbon utilization of *Xag* (**Figure 6**). Therefore, we investigated the biofilm formation abilities and EPS production of *Xag*(EV), *Xag*(LcrX), *Xag*∆*lcrX*(EV), and *Xag*∆*lcrX*(LcrX^P^). Because we used XVM2 medium for the assays, the growth patterns of the four strains were measured in the medium (**Supplementary Figure S7**). The four strains showed similar growth patterns, although *Xag*(LcrX) was slightly low in only 48 h after incubation. Biofilm formation was measured in a 96-well PVC plate assay. The biofilm formation abilities significantly differed between *Xag*(LcrX) and *Xag*∆*lcrX*(EV) (**Figure 7A**). Biofilm formation by *Xag*(LcrX) was reduced (1.3-fold), while that by *Xag*∆*lcrX*(EV) was significantly increased (1.8-fold) compared to that of *Xag*(EV). Biofilm formation of the complemented strain, *Xag∆lcrX*(LcrX^P^), was restored to the level of *Xag*(EV), indicating that *Xag∆lcrX* was functionally complemented by LcrX^P^. Because bacterial cells produce EPS, a component of biofilm (Sutherland, 2001), we examined whether the change in biofilm formation was affected by EPS production. However, unlike the biofilm formation patterns (**Figure 7A**), which were reduced in *Xag*(LcrX) and increased in *Xag∆lcrX(*EV), there were no significant differences in EPS production between the four strains (**Figure 7B**). These results indicate that LcrX is involved in biofilm formation but not in EPS production in *Xag*. Additionally, bacterial motility is known to be closely involved in biofilm formation because flagella contribute to *the surface attachment* (Lemon et al., 2007)*. Therefore, we examined bacterial motility using two different assays* (**Figure 8**). The swarming motility in *Xag*(LcrX) and *Xag∆lcrX*(EV) was not different from this in *Xag*(EV) (**Figures 8A** and **8B**). However, the swimming halo in *Xag*(LcrX) was reduced compared with Xag(EV) (**Figures 8C** and **8D**). *XagΔlcrX*(EV) tended to enhance the swimming motility compared with Xag(EV). The complemented strain was comparable with *Xag*(EV). These data suggest that LcrX affects the flagella motility in liquid or inside low-viscosity conditions.

### Overexpression of LcrX Reduced Siderophore Secretion

**Figure 9:**
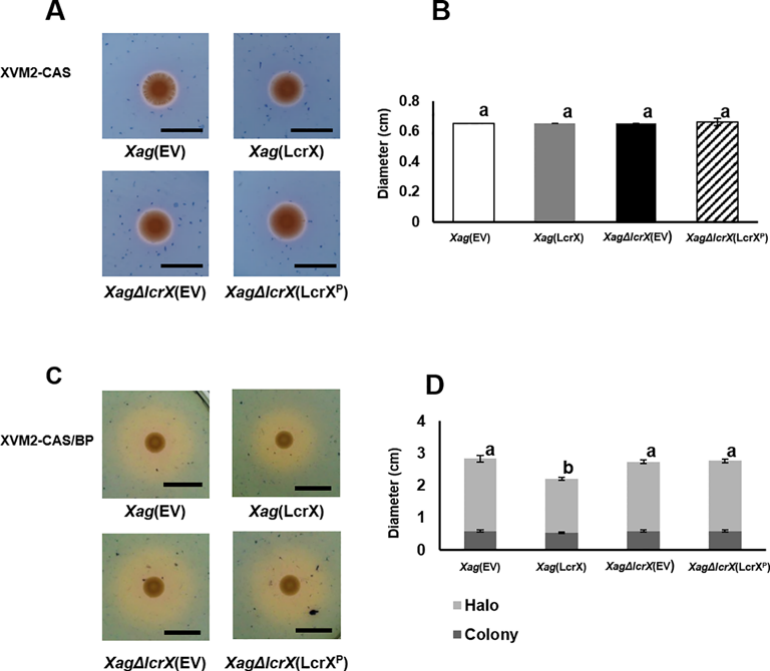
Chrome azurol S (CAS) assay of *Xag*(EV), *Xag*(LcrX), *XagΔlcrX*(EV), and *XagΔlcrX*(LcrX^P^). Three microliters of a bacterial suspension at OD_600_ of 0.3 were dropped on **(A** and **B)** the iron-rich medium, XVM2-CAS, and **(C** and **D)** iron-limited medium, XVM2-CAS-BP which contained 2,2’-bipyrudyl (BP). **(A** and **C)** Photographs of colony and halos were taken **(B** and **D)**, and their diameters were measured at 3 days after incubation. Black bars in the photographs represent 1 cm. Error bars indicate the means of five biological replicates with the standard deviation. Different letters on the error bar graph represent significant differences by one-way ANOVA (p < 0.05). This experiment was repeated at least three times.

Proteomic analysis showed that diverse iron-related proteins, including TonB-dependent receptors, were less abundant in *Xag*(LcrX) compared to in *Xag*(EV) (**Supplementary Tables S4** and **S5**). TonB-dependent receptors are part of the iron-uptake system, which helps bacteria absorb this mineral under iron-limited conditions (Schauer et al., 2008; Noinaj et al., 2010). Therefore, a CAS assay was performed to evaluate siderophore secretion. As shown in **Figures 9A** and **9B**, no obvious difference was found between the four strains under the iron-rich condition. However, under the iron-limited condition, the diameter of the yellow halo, which represents siderophore secretion, was reduced (approximately 70%) in *Xag*(LcrX) compared with *Xag*(EV) (**Figures 9C** and **9D**). These data indicate that overexpression of LcrX in *Xag* diminished siderophore production under the iron-limited condition, but not under the iron-rich condition.

## Discussion

In many prokaryotes, gene expression is controlled by diverse TFs to acclimate to new environments. Among the TF families, the LTTR family is highly conserved in bacteria (Rivera-Gomez et al., 2011). A previous study reported that the N-terminus of LTTRs contains an HTH motif as a DNA-binding domain, and the C-terminus possesses an activation domain (Schell, 1993; Zaim and Kierzek, 2003). The predicted 3D structure of LcrX also shows two domains, a DNA-binding domain, and LysR activation domain, suggesting that LcrX belongs to the LTTR family. The low homology with OxyR, CcmR, and CynR (**Figure 1C**) suggests that LcrX is involved in different mechanisms from those transcriptional regulators. LTTR is the largest family and found not only in *Xanthomonas* spp., but also in other bacteria and even in fungi (Minezaki et al., 2005). Previous studies reported that the LTTRs are mainly involved in amino acid metabolisms such as IlvY for isoleucine/valine biosynthesis and ArgP for arginine transport in *E. coli.* (Wek and Hatfield, 1988; Marbaniang and Gowrishankar, 2011). In addition to amino acid metabolism, LTTRs are also associated with carbohydrate metabolism, quorum sensing, cell division, and virulence (Sheehan and Dorman, 1998; Cao et al., 2001; Jones et al., 2003). Therefore, LTTRs may not be restricted to one biological mechanism in bacteria and likely control diverse cellular and metabolic processes, contributing to their phenotypic alteration ability and virulence.

In the EMSA, we found that LcrX directly bound to the putative promoter of protease (AOY62452), which was more (over 3-fold) abundant in *XagΔlcrX*(EV) compared to in *Xag*(EV) and in *Xag*(EV) compared with *Xag*(LcrX). These data suggest that protease was negatively regulated by LcrX. However, extracellular protease activity was not changed in the mutants. The protease, AOY62452 showed high homology with EcpA (92%, 335/362), which encodes an extracellular protease in *Xoc* (Zou et al., 2012). *Xoc* lacking EcpA did not show the extracellular protease activity. Interestingly, extracellular protease activity by EcpA was observed in *Xoc*, whereas this activity was not observed in *Xoo*, although the two pathovars infect the same host. A comparison of EcpA from *Xoo* and *Xoc* revealed that 1–282 aa are nearly identical, while 283–366 aa are completely different. Furthermore, the protease activity was observed when the N-terminal of *Xoo* EcpA was fused with the C-terminal of *Xoc* EcpA, but not vice versa (Zou et al., 2012), indicating that the C-terminal regions are indispensable for protease activity. Comparison of the amino acid sequence between AOY62452 and EcpA of *Xoc* revealed that the C-terminal region residues (264–296 aa) of AOY62452 differed from those of *Xoc* EcpA (data not shown). Therefore, the functions of AOY62452 may differ from those of EcpA, and the extracellular protease activity of *Xag*(LcrX) was not changed, although LcrX negatively regulates AOY62452 expression. Additionally, *Xag* possesses more than 26 proteins, including 5 proteases, 10 serine proteases, 4 ATP-dependent proteases, 5 metalloproteases, a zinc-dependent protease, a cysteine protease, and over 90 peptidases. The extracellular protease activity of *Xag* under the given condition may have been affected by other proteases and peptidases.

Interestingly, overexpression of LcrX led to reduced virulence and biofilm formation. Biofilm formation is a major virulence factor because it allows bacteria to protect themselves under stressful conditions, including antimicrobial activity from the host (Costerton et al., 1999; Donlan and Costerton, 2002; Luppens et al., 2002). *Escherichia coli* and *Vibrio cholerae* strains with impaired biofilm formation abilities were less virulent compared with their wild-type strains (Zhu et al., 2002; Naves et al., 2008; Huang et al., 2013). In agreement with these previous studies, the reduced biofilm formation of *Xag*(LcrX) may contribute to virulence in *Xag*. In the proteomic analysis, the expression of proteins related to carbohydrate metabolism was affected by LcrX. Carbohydrates are one of the main components of biofilm. For example, GlpG is a glycogen phosphorylase involved in glycogen metabolism. An *Azospirillum brasilense* strain deficient in GlpG had a different monosaccharide composition in extracted EPS and significantly reduced biofilm formation ability compared to the wild-type strain (Lerner et al., 2009). This study demonstrates that glycogen regulation is involved in biofilm formation and sugar metabolic pathways. Therefore, LcrX may be involved in biofilm formation by regulating carbohydrate metabolism. In addition, proteomic data showed that proteins related to signal transduction mechanisms, including histidine kinases, response regulators, and c-di-GMP phosphodiesterase were abundantly in *Xag*(EV) vs. *Xag*(LcrX). It is very well-known that these types of proteins function to control biofilm formation. For example, in *Streptococcus mutans*, which lacks histidine kinase and response regulators, biofilm formation and acid tolerance were reduced (Li et al., 2002). Similarly, the histidine kinase BinK negatively regulates biofilm formation in *Vibrio fischeri* (Brooks and Mandel, 2016). Additionally, overexpression of RbdA, which regulates c-di-GMP phosphodiesterase, resulted in reduced biofilm formation, whereas biofilm formation was significantly increased in *P. aeruginosa* cells lacking RdbA (An et al., 2010). LcrX may be a transcriptional regulator that directly or indirectly controls the expression of proteins related to carbohydrate metabolism and signal transduction pathways and regulates biofilm formation as well as virulence. Additionally, it is known that the swimming motility is closely related to an attachment of surfaces in a high humidity condition, biofilm formation, and virulence (Pratt and Kolter, 1998; Tans-Kersten et al., 2004; Zhu et al., 2019). We also showed that swimming motility in *Xag*(LcrX) was decreased. Therefore, it can be postulated that the reduction of the swimming motility is one of the reasons for the low biofilm in *Xag*(LcrX), which may contribute to virulence in *Xag*.

Particularly, iron-associated proteins such as bacterioferritin, ferrous iron transporter B, and TonB-dependent receptors, which are outer membrane proteins and siderophore receptors (Turner et al., 2001), were abundant in *Xag*(EV) compared with *Xag*(LcrX). The iron uptake system is important in bacteria because iron is essential for energy generation and cell growth (Skaar, 2010). Therefore, bacteria must produce iron-chelators, called siderophores, to absorb iron (Fe^3+^) from the environment (Neilands, 1995). According to recent reports, the roles of siderophores in virulence differ in *Xanthomonas* spp. For example, siderophores in *Xoo*, the causal agent of bacterial leaf blight disease on rice, which mainly colonizes the xylem vessel and is not essential for virulence, but siderophores from *X. oryzae* pv. *oryzicola* (*Xoc*), which causes bacterial streak disease on rice and colonizes the intercellular space in the mesophyll, are required for optimum virulence (Pandey and Sonti, 2010; Rai et al., 2015; Pandey et al., 2016). In addition, the virulence of *X*. *campestris* pv. *campestris*, which is a causal agent of black rot disease in crucifers, was reduced when the siderophore synthesis gene (*xssA*) was deficient (Pandey et al., 2017). In this study, we showed that secretion of siderophores from the LcrX-overexpressing strain was dramatically reduced compared to that from the wild-type strain, suggesting that overexpression of LcrX negatively regulates siderophore production. This may explain the reduced virulence of *Xag*(LcrX).

Proteomic analysis revealed that many proteins in G (carbohydrate metabolism and transport) were differentially abundant between *Xag*(EV) and *Xag*(LcrX), and the overexpression of LcrX led reduced biofilm formation consisting of polysaccharides and other biomolecules. Based on these results, we hypothesized that LcrX is involved in regulating carbohydrate metabolism. Indeed, LcrX directly bound to the putative promoter of FBP, which possesses putative LTTR boxes (**Supplementary Figure S8**). FBP was more (over 5.3-fold) abundant in *Xag*(EV) compared to in *Xag*(LcrX), suggesting that LcrX negatively regulates FBP expression. Previous studies reported that FBP and fructose-bisphosphate aldolases, whose substrates are fructose-bisphosphate, are involved in the use of carbon sources and virulence. *Mycobacterium marinum*, lacking GlpX that encodes a fructose 1, 6-bisphosphatase, showed reduced growth in the presence of the gluconeogenic carbon source and was less virulent in *Zebrafish* (Tong et al., 2016). Additionally, fructose-bisphosphate aldolase-deficient mutants of *Xoc* and *Francisella novicida* displayed growth retardation in various carbon sources and showed reduced virulence in their hosts (Guo et al., 2012; Ziveri et al., 2017).

Proteomic analysis revealed that six and two proteins belonging to the group G (carbohydrate metabolism) were more abundant in *Xag*(LcrX) and *XagΔlcrX*(EV), respectively, compared to in *Xag*(EV) (**Supplementary Tables S5** and **S7**). In addition, the GEN III plate assay at 48 h after of incubation showed that the growth of *Xag*(LcrX) was reduced under 18 different conditions, which contained different carbon sources and not chemical substances, compared to the growth of *Xag*(EV), suggesting that LcrX is related to the regulation of carbohydrate metabolism Furthermore, the proteomic analysis and EMSA results support the roles of LcrX in regulating carbohydrate metabolism. Therefore, overexpression of LcrX may trigger growth retardation by negatively regulating carbohydrate metabolism. Bacterial growth patterns in the presence of sugar sources as a sole carbon source showed a similar pattern as the GEN III plate assay. The growth of *Xag*(LcrX), but not *XagΔlcrX*(EV), was reduced compared to that of *Xag*(EV). Collectively, these data demonstrate that under the solo carbon condition, overexpressed LcrX negatively regulates bacterial growth and causes growth retardation in *Xag*. Finally, these different abilities to use diverse carbon sources by *Xag*(LcrX) and *XagΔlcrX*(EV) may explain why *Xag*(LcrX) was less virulent, while *XagΔlcrX*(EV) was not.

In this study, the functions of LcrX in *Xag* were postulated by using the predicted 3D structure and label-free shotgun comparative proteomics combined with COG categorization. We further characterized the functions of LcrX through diverse phenotypic assays, including the GEN III plate assay. Taken together, LcrX is involved in siderophore production, biofilm formation, and carbohydrate metabolism, but not in EPS production and motility, which may contribute to its virulence in *Xag*. Finally, we demonstrated that LcrX binds to specific putative promoters containing LTTR boxes. This study provides fundamental and valuable information regarding the biological functions of a previously uncharacterized transcriptional regulator in plant pathogenic bacteria.

## Data Availability Statement

The mass spectrometry proteomics data have been deposited to the ProteomeXchange Consortium *via* the PRIDE (Perez-Riverol et al., 2019) partner repository with the dataset identifier PXD016274.

## Author Contributions

S-WH conceived the study. S-WH and HP designed the experiments. HP, MK, H-JP, and JL conducted the experiments. ED conducted EMSA. HP and S-WH analyzed the data and prepared the manuscript. All authors reviewed the manuscript.

## Conflict of Interest

The authors declare that the research was conducted in the absence of any commercial or financial relationships that could be construed as a potential conflict of interest.
