# Supplementary figures and images for: A LysR-Type Transcriptional Regulator LcrX Is Involved in Virulence, Biofilm Formation, Swimming Motility, Siderophore Secretion, and Growth in Sugar Sources in Xanthomonas axonopodis Pv. glycines
Source: Front Plant Sci. 2020 Jan 10;10:1657. doi: 10.3389/fpls.2019.01657 (PMC6965072; doi:10.3389/fpls.2019.01657)

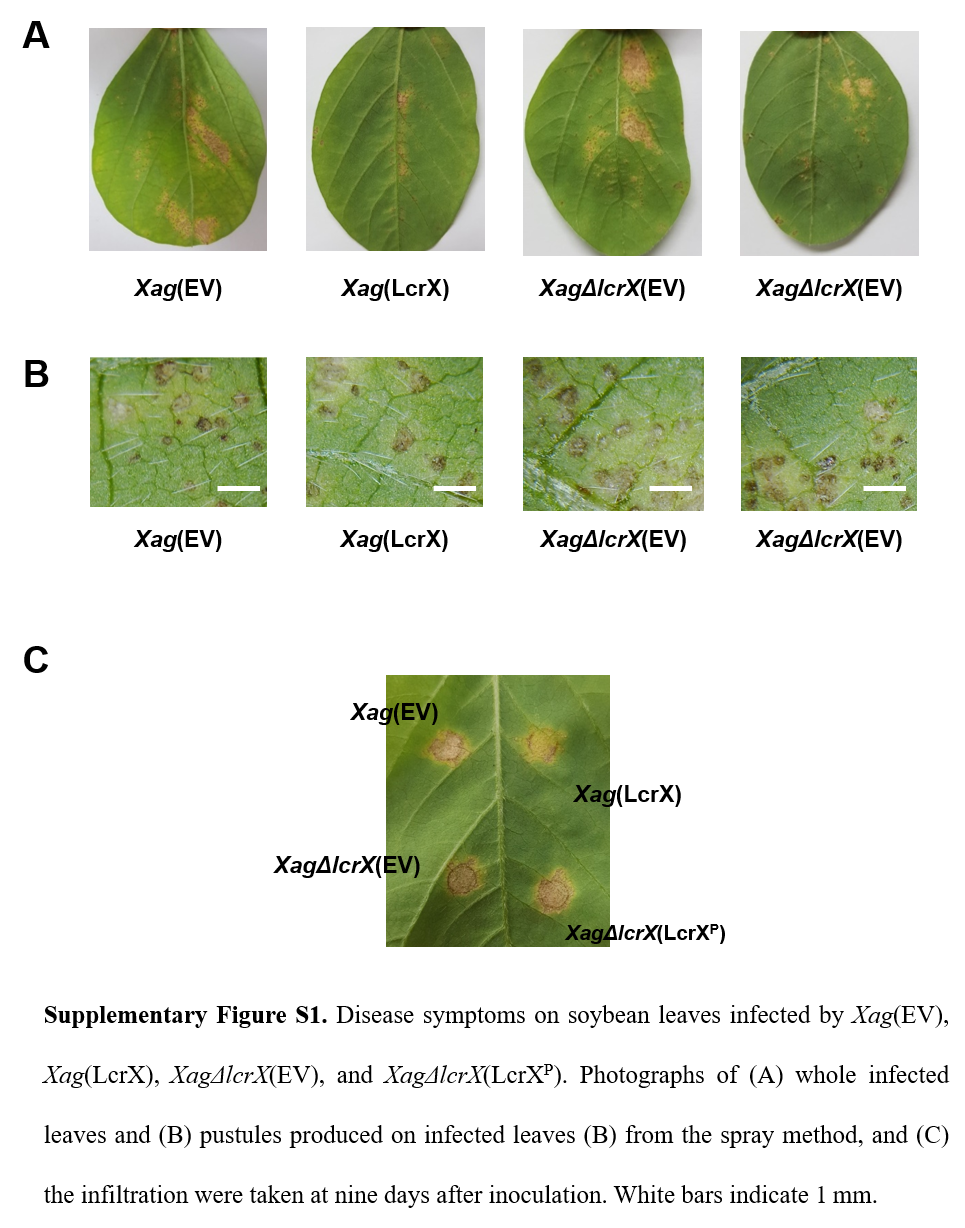

Supplement: Supplementary file 1 [file Image_1.tiff]

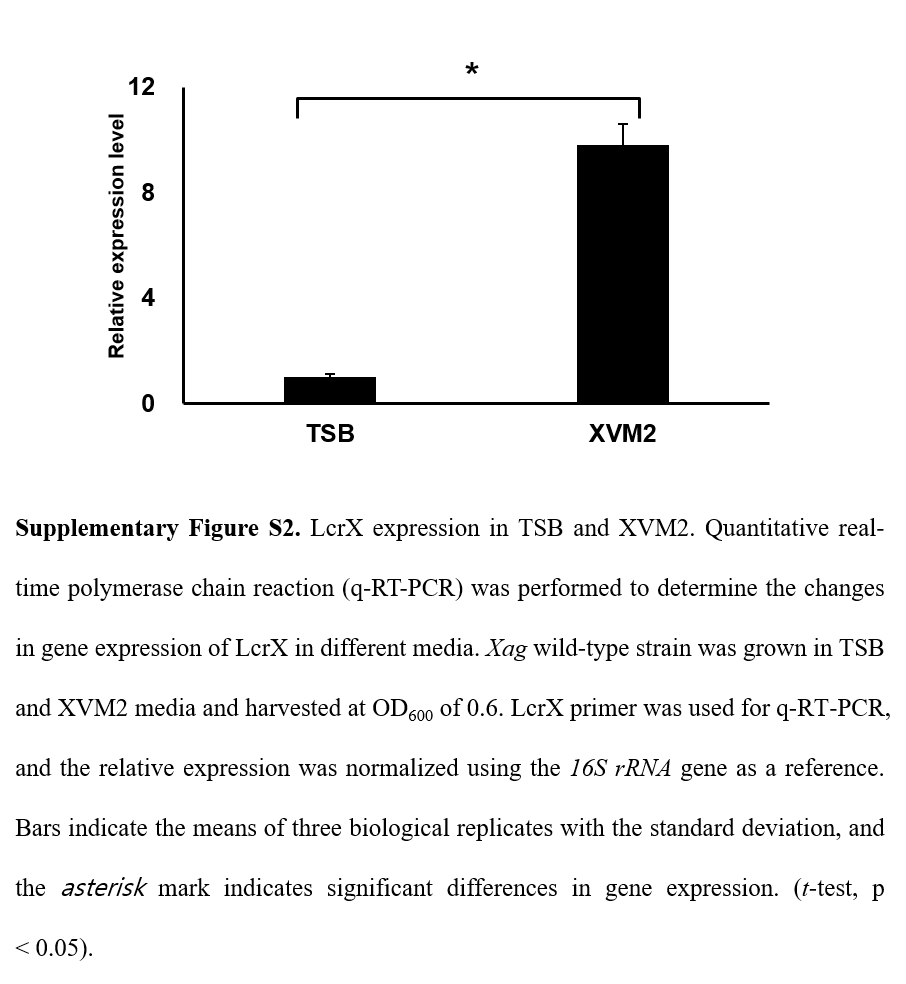

Supplement: Supplementary file 2 [file Image_2.tiff]

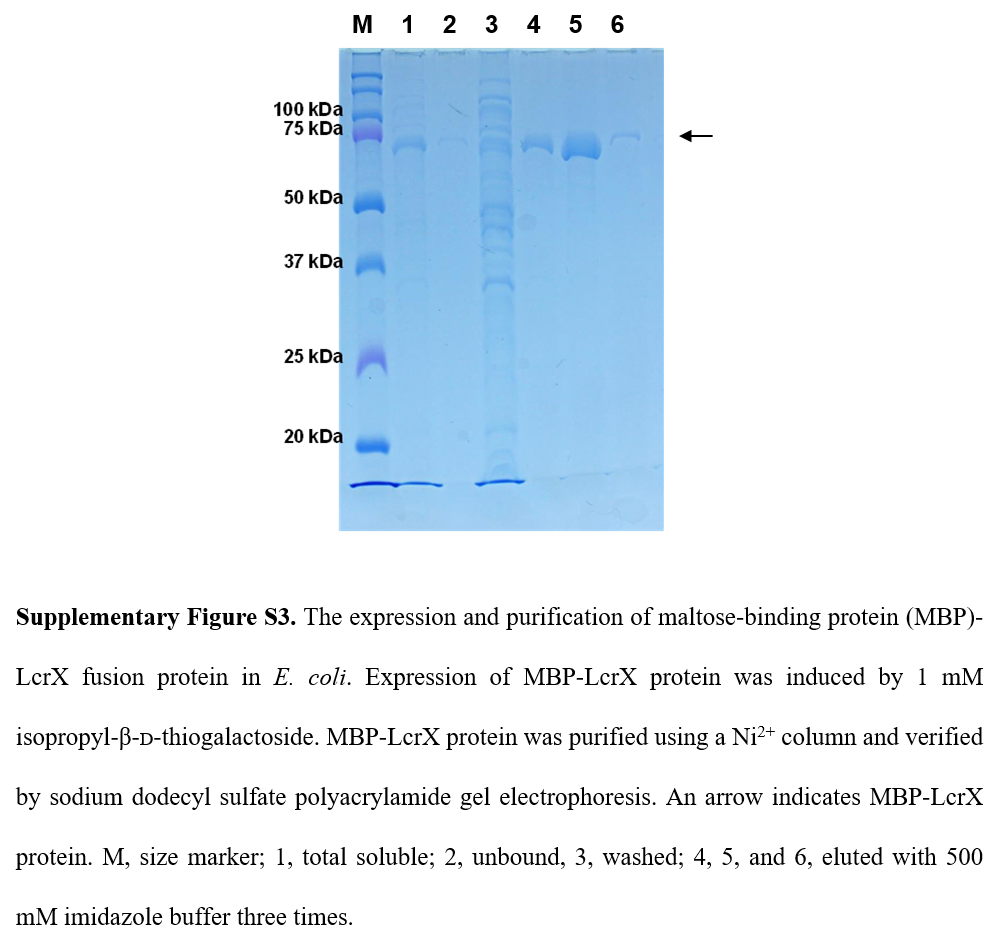

Supplement: Supplementary file 3 [file Image_3.tiff]

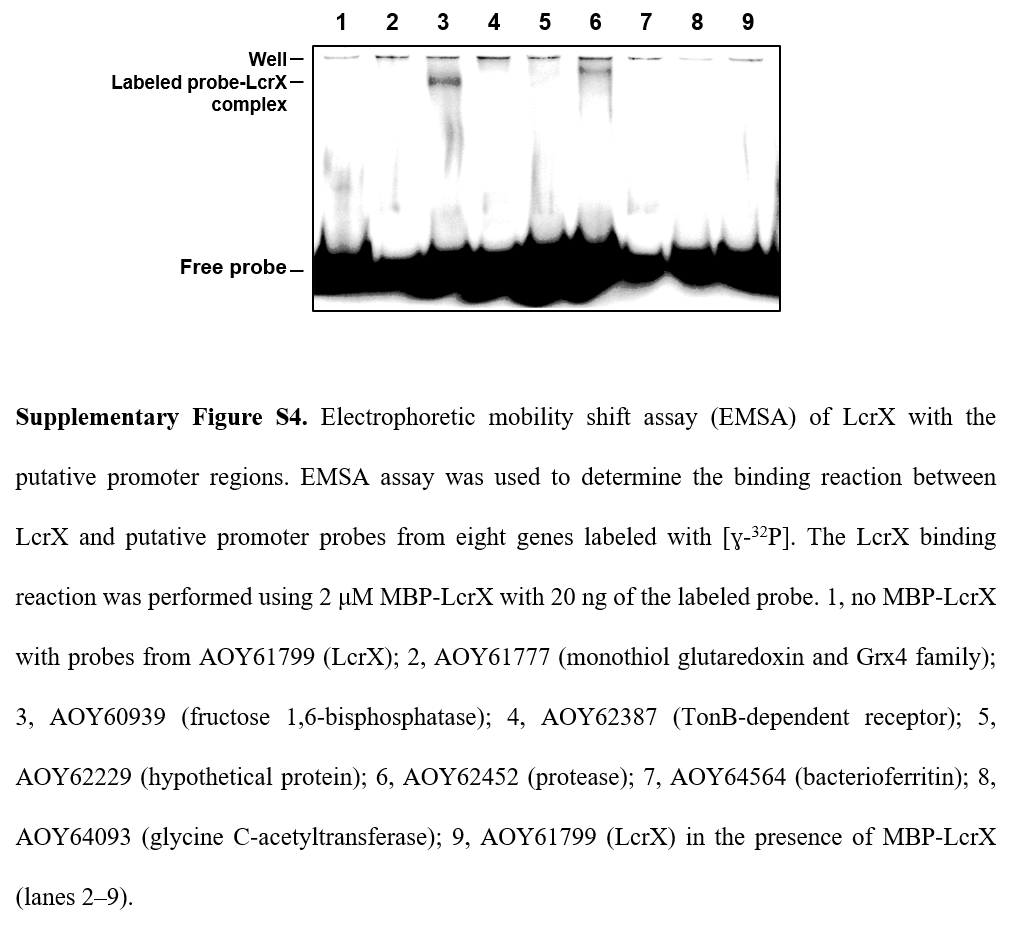

Supplement: Supplementary file 4 [file Image_4.tiff]

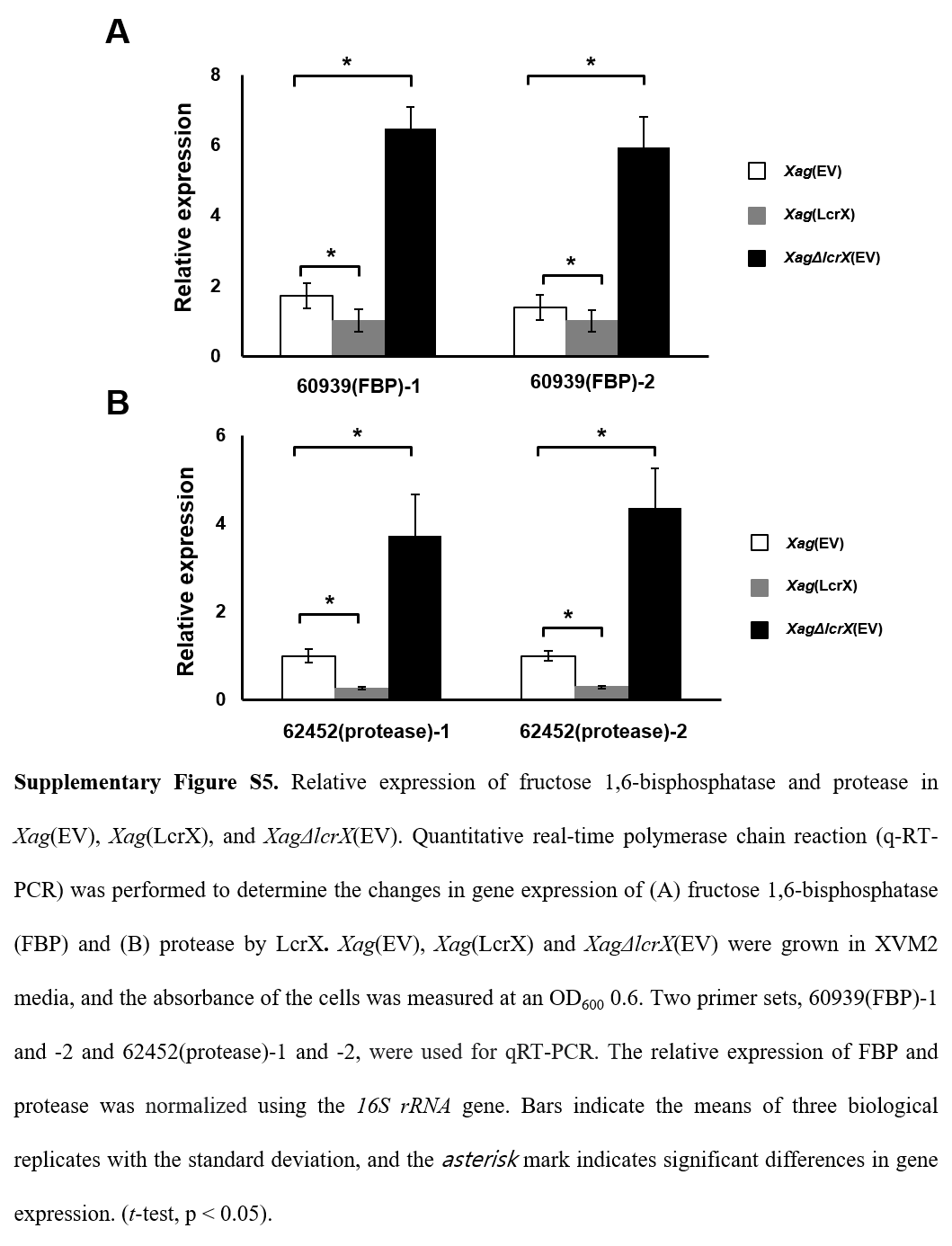

Supplement: Supplementary file 5 [file Image_5.tiff]

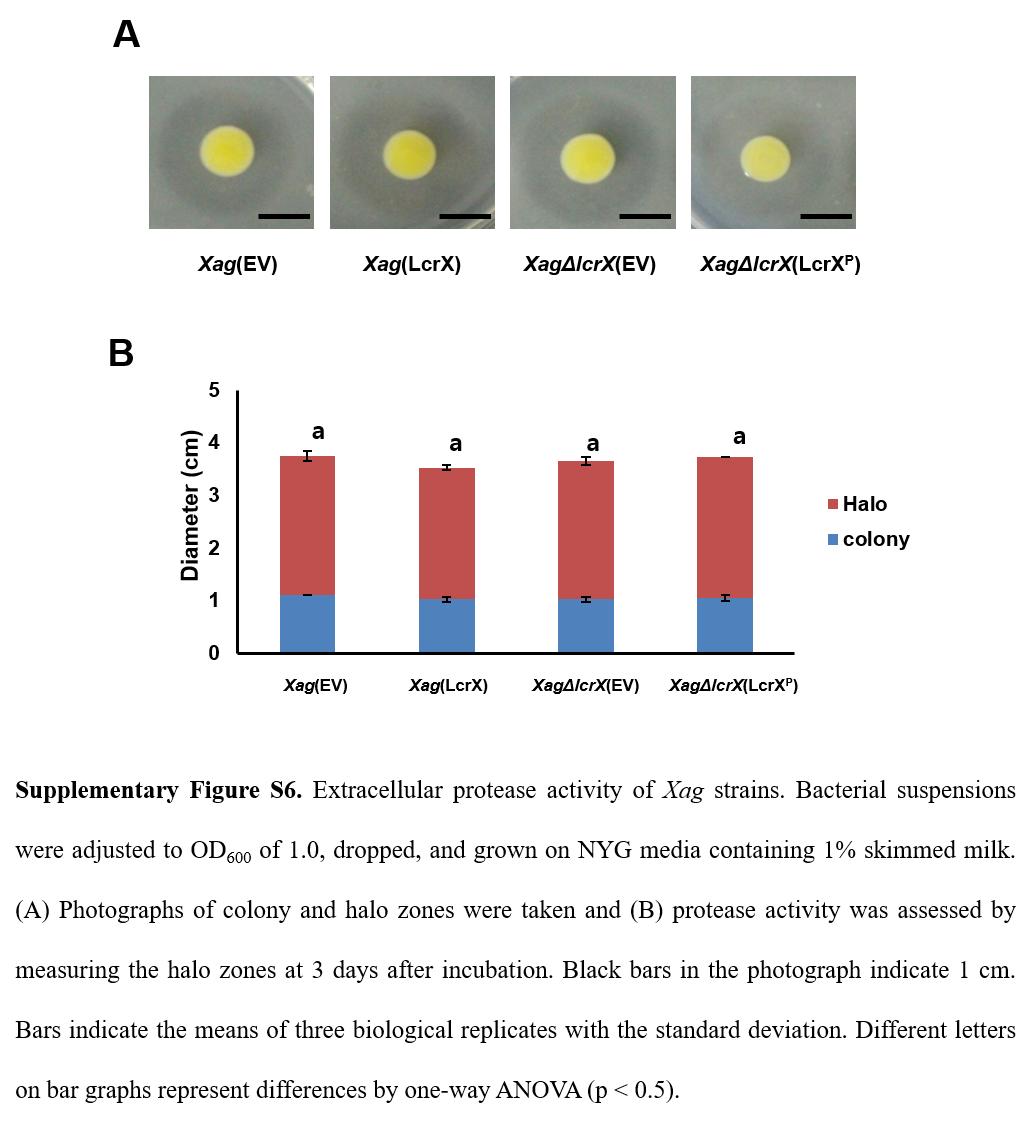

Supplement: Supplementary file 6 [file Image_6.tiff]

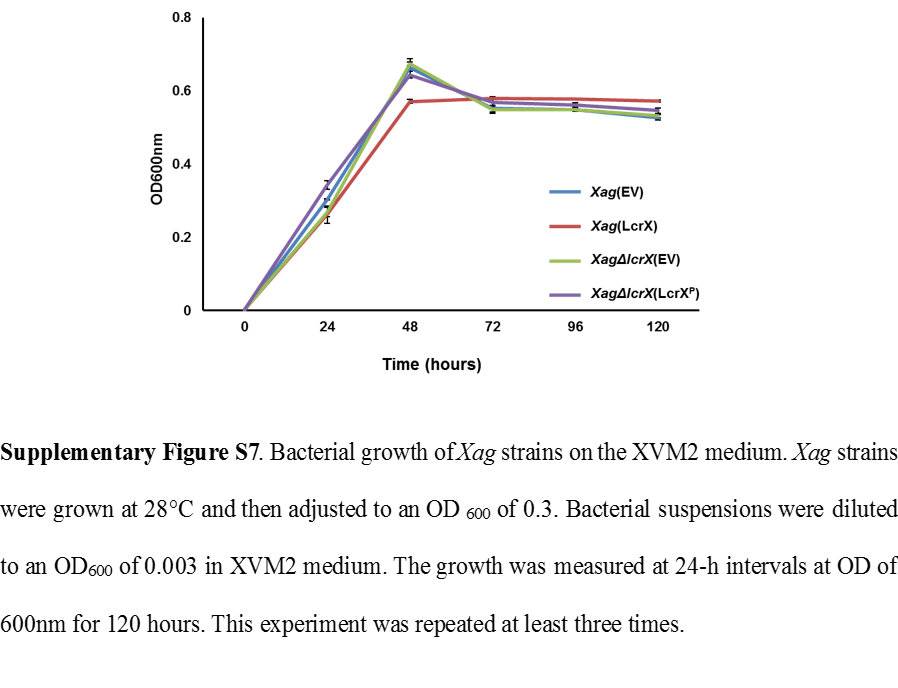

Supplement: Supplementary file 7 [file Image_7.tiff]

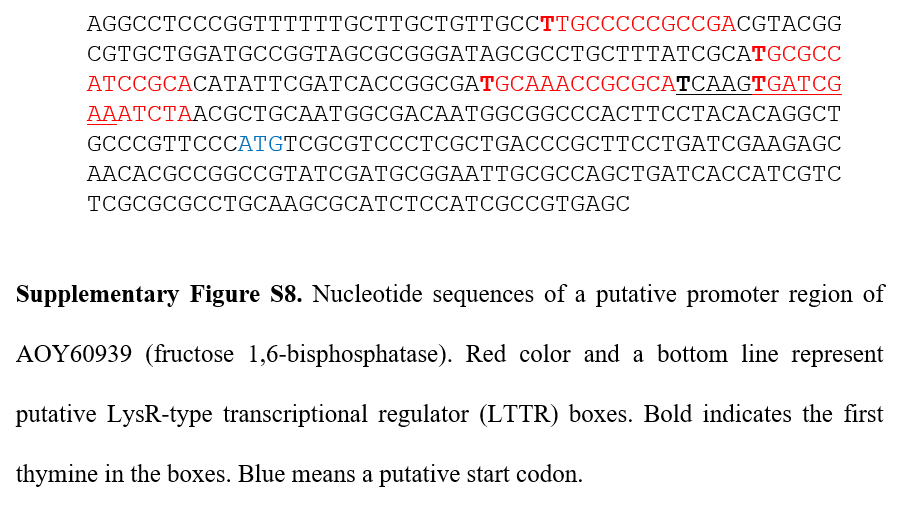

Supplement: Supplementary file 8 [file Image_8.tiff]
