# Supplementary material for: A LysR-Type Transcriptional Regulator LcrX Is Involved in Virulence, Biofilm Formation, Swimming Motility, Siderophore Secretion, and Growth in Sugar Sources in Xanthomonas axonopodis Pv. glycines
Source: Front Plant Sci. 2020 Jan 10;10:1657. doi: 10.3389/fpls.2019.01657 (PMC6965072; doi:10.3389/fpls.2019.01657)
Supplement: Supplementary file 9 [file Table_1.docx]

**Table S1. Bacteria strains and plasmids used in this study**

| **Strains and plasmids** | | | | | | | | **Characteristics** | **Source or references** | | | | | | |
| --- | --- | --- | --- | --- | --- | --- | --- | --- | --- | --- | --- | --- | --- | --- | --- |
| *Xanthomonas axonopodis* pv*. glycine* | | | | | | | | | | | | |  |  |  |
| 8ra | | | | Wild type, Cp^r^ | | | | | Seong et al., 2016 | | |  |  |  |  |
| *Xag*(EV) | | | | Wild type carrying pBBR1-MCS5, Gm^r^ | | | | | This study | | |  |  |  |  |
| *Xag*(LcrX) | | | | Wild type carrying pBBR1LcrX, Gm^r^ | | | | | This study | | |  |  |  |  |
| *XagΔlcrX*(EV) | | | | Insertional mutant in *lcrX* carrying pBBR1-MCS5, Km^r^ and Gm^r^ | | | | | This study | | |  |  |  |  |
| *XagΔlcrX*(LcrX^P^) | | | | *XagΔLcrX* complemented strain with pBBR1LcrX^P^, Km^r^ and Gm^r^ | | | | | This study | | |  |  |  |  |
| *Escherichia coli* | | | | |  |  |  |  |  |  |  |  |  |  |  |
| DH5α | | | | F^–^, `80*dlaczZΔM15*, *Δ*(*lacZY*A‐argF), U169, *deoR*, RecA1, *end*A1, *hsd*R17, *gyr*A96, *thit‐*1, *rel*A1, supE44 | | | | | Promega | | |  |  |  |  |
| BL21(DE3) pLys | | | | F^–^, *omp*T, *hsd*SB (r_B_–, m_B_–), *dcm*, *gal*, λ(DE3), pLysS | | | | | Promega | | |  |  |  |  |
| BL21(MBP-LcrX) | | | | BL21(DE3) pLys carrying pOPINM-LcrX | | | | | This study | | |  |  |  |  |
| Plasmids | | | | |  |  |  |  |  |  |  |  |  |  |  |
| pGEM-T Easy | | | | Vector for TA cloning, Am^r^ | | | | | Promega | | |  |  |  |  |
| pGEM-lcrX | | | | pGEM-T Easy inserted by 1301-bp *lcrX* fragment by TA ligation, Am^r^ | | | | | This study | | |  |  |  |  |
| pUC4K | | | | Vector carrying kanamycin cassette, Km^r^ | | | | | Pharmacia | | |  |  |  |  |
| pGEM-lcrX::KM | | | | A derivative of pGEM-IcrX. *lcrX* gene disrupted by kanamycine cassette, Am^r^, Km^r^ | | | | | This study | | |  |  |  |  |
| pBBR1MCS-5 | | | | Broad-host-range vector, *lacZ* promoter, Gm^r^ | | | | | Kovach et al., 1995 | | |  |  |  |  |
| pBBR1MCS-5^P^ | | | | A derivative of pBBR1-MCS5. *lacZ* promoter(76 bp) was deleted, Gm^r^ | | | | | This study | | |  |  |  |  |
| pGEMLcrX-OE | | | | pGEM-T Easy carrying open reading frame (903 bp) of *lcrX*, containing 6xHis-tag at N-terminus at backward primer by TA ligation, Am^r^ | | | | | This study | | |  |  |  |  |
| pGEMLcrX^P^ | | | | pGEM-T Easy carrying a putative promoter region (388 bp) and open reading frame (903 bp) of *lcrX*, containing 6xHis-tag at N-terminus at backward primer by TA ligation, Am^r^ | | | | | This study | | |  |  |  |  |
| pBBR1LcrX | | | | pBBR1-MCS5 carrying a 903-bp with 6xHis-tag at N-terminus from pGEMLcrX-OE, Gm^r^ | | | | | This study | | |  |  |  |  |
| pBBR1LcrX^P^ | | | | pBBR1-MCS5 carrying a 1291-bp with 6xHis-tag at N-terminus from pGEMLcrX^P^, Gm^r^ | | | | | This study | | |  |  |  |  |
| pOPINM | | | | Vector for protein expression, contain 6xHis-tag and MBP at N-terminus, Am^r^ | | | | | Berrow et al., 2007 | | |  |  |  |  |
| pOPINM-LcrX | | | pOPINM carrying open reading frame (903 bp) of *lcrX*, Am^r^ | | | | | | This study | | | | | | |
