# Supplementary material for: A LysR-Type Transcriptional Regulator LcrX Is Involved in Virulence, Biofilm Formation, Swimming Motility, Siderophore Secretion, and Growth in Sugar Sources in Xanthomonas axonopodis Pv. glycines
Source: Front Plant Sci. 2020 Jan 10;10:1657. doi: 10.3389/fpls.2019.01657 (PMC6965072; doi:10.3389/fpls.2019.01657)
Supplement: Supplementary file 10 [file Table_2.docx]

**Table S2. Primers used in this study**

| **Primer** | | **Sequence (5’ to 3’)** | |  |  |
| --- | --- | --- | --- | --- | --- |
| **Construct for pGem-lcrX::KM for LcrX knockout strain** | | | |  |  |
| *XagΔlcrX* -F | | CGATGCAAGTGCTGGCTTTT | | |  |
| *XagΔlcrX* -R | | AAGAAGATTCCGCGTCTCCC | | |  |
| **Construct for pBBR1LcrX** | | | | |  |
| *Xag*(LcrX)-F | | GTCGACATGACCATCAAATTCGACC | | |  |
| *Xag*(LcrX)-R | | GGATCCTCAGTGGTGGTGGTGGTGGTGCCTTGAACGTGCGGGCATCC | | |  |
| MCS5-F | | ATGCTTCCGGCTCGTATG | | |  |
| MCS5-R | | CAGGGTTTTCCCAGTCACGA | | |  |
| **Construct for pBBR1LcrX^P^** | | | | |  |
| MCS-5^P^-F | | GCCTGGGGTGCCTAATGAGTG | | |  |
| MCS-5 ^P^-R | | GACCATGATTACGCCAAGCG | | |  |
| *Xag*(LcrX ^P^)- F | | GTCGACAATGGCGAGACGCGCAGGC | | |  |
| **LcrX purification and EMSA** | | | | |  |
| MBP-*Xag*(LcrX)-F | | AAGTTCTGTTTCAGGGCCCGACCATCAAATTCGACCTCAA | | |  |
| MBP-*Xag*(LcrX)-R | | ATGGTCTAGAAAGCTTTATCACCTTGAACGTGCGGGCA | | |  |
| AOY61799-F | | GCCGATGCAGCGCCATCGCA | | |  |
| AOY61799-R | | GGTGCCGCAGCTTCAGTGTT | | |  |
| AOY62229-F | | TGGAGCAAGCGGATGCTGTA | | |  |
| AOY62229-R | | ACGATGTCCTGTCCTTTGGT | | |  |
| AOY62387-F | | CCTTCTTCCAAATGCAGAAG | | |  |
| AOY62387-R | | GCGCAAGGCTCCTTGTCTTG | | |  |
| AOY64564-F | | GCGCTGGAGCTACCTGCAGC | | |  |
| AOY64564-R | | GCGATGTCTCCGAGAGAGTG | | |  |
| AOY61777-F | | CAGCGCATGCGACAGCAGCA | | |  |
| AOY61777-R | | GGCAGCACTTCGGTGGCAGC | | |  |
| AOY60939-F | | AGGCCTCCCGGTTTTTTGCT | | |  |
| AOY60939-R | | GGGAACGGGCAGCCTGTGTA | | |  |
| AOY62452-F | | TGCCAGTCGTTGACACATTC | | |  |
| AOY62452-R | | CAGTAACTCTCCGTGTGATC | | |  |
| AOY64093-F | | GAAAAGAAAGGAGGTAAAAC | | |  |
| AOY64093-R | | GGCGGTCGCAATGGCAGAAA | | |  |
| **qPCR** | |  | | |  |
| 16S-F | | AGTTCGCATCGTTTAGGG | | |  |
| 16S-R | | GACCAACACTGACACTGAG | | |  |
| LcrX-F | | GACTCGAGCTTGTCGATCCT | | |  |
| LcrX-R | | ATTCGACCTCAACGATCTGC | | |  |
| AOY60939-1F | | CAGCGCATGTTGAAGTCCT | | |  |
| AOY60939-1R | | CCATCAACATGTCCAACCAA | | |  |
| AOY60939-2F | | GGTTGGCTTCGTACATCAGG | | |  |
| AOY60939-2R | | GACTTCAACATGCGCTGGAT | | |  |
| AOY62452-1F | | CATTGACCATCGAACTGTCG | | |  |
| AOY62452-1R | | CCAGAATGCCGTTATCCAGT | | |  |
| AOY62452-2F | | TGAGTCACTTCACGGTCGTC | | |  |
| AOY62452-2R | | TGCGAAGTACTCATGGCTGT | | |  |
