# Supplementary material for: A LysR-Type Transcriptional Regulator LcrX Is Involved in Virulence, Biofilm Formation, Swimming Motility, Siderophore Secretion, and Growth in Sugar Sources in Xanthomonas axonopodis Pv. glycines
Source: Front Plant Sci. 2020 Jan 10;10:1657. doi: 10.3389/fpls.2019.01657 (PMC6965072; doi:10.3389/fpls.2019.01657)
Supplement: Supplementary file 11 [file Table_3.docx]

**Table S3. Detected proteins and peptide spectral matches (PSM) from *Xag*(EV) vs. *Xag*(LcrX) and *Xag*(EV) vs. *XagΔlcrX*(EV) in three biological replicates from Liquid chromatography-mass spectrometry.**

| **Strain** | **1st** | | **2st** | | **3st** | | **Shared protein in 3 biological replicates** |
| --- | --- | --- | --- | --- | --- | --- | --- |
|  | **Protein** | **PSM** | **Protein** | **PSM** | **Protein** | **PSM** |  |
| ***Xag*(EV) ^#^** | **1248** | **73155** | **1280** | **73203** | **1292** | **72986** | **1178** |
| ***Xag*(LcrX)^#^** | **1269** | **78,699** | **1285** | **78,668** | **1257** | **78,534** | **1119** |
| ***Xag*(EV)*** | **1160** | **65499** | **1159** | **65450** | **1149** | **65202** | **1091** |
| ***XagΔlcrX*(EV)*** | **1187** | **67089** | **1157** | **67168** | **1161** | **65723** | **1097** |

^#,^ * The same symbols on strains were simultaneously analyzed.
